# Structural and functional characterization of the IgSF21-neurexin2α complex and its related signaling pathways in the regulation of inhibitory synapse organization

**DOI:** 10.3389/fnmol.2024.1371145

**Published:** 2024-03-20

**Authors:** Nicolas Chofflet, Yusuke Naito, Anthony John Pastore, Nirmala Padmanabhan, Phuong Trang Nguyen, Christian Poitras, Benjamin Feller, Nayoung Yi, Jeremie Van Prooijen, Husam Khaled, Benoit Coulombe, Steven J. Clapcote, Steve Bourgault, Tabrez J. Siddiqui, Gabby Rudenko, Hideto Takahashi

**Affiliations:** ^1^Synapse Development and Plasticity Research Unit, Institut de Recherches Cliniques de Montréal, Montreal, QC, Canada; ^2^Integrated Program in Neuroscience, McGill University, Montreal, QC, Canada; ^3^Department of Pharmacology and Toxicology, Sealy Center for Structural Biology and Molecular Biophysics, University of Texas Medical Branch, Galveston, TX, United States; ^4^PrairieNeuro Research Centre, Health Sciences Centre, Kleysen Institute for Advanced Medicine, Winnipeg, MB, Canada; ^5^Department of Physiology and Pathophysiology, University of Manitoba, Winnipeg, MB, Canada; ^6^Quebec Network for Research on Protein Function, Engineering and Applications (PROTEO), Department of Chemistry, Université du Québec à Montréal, Montreal, QC, Canada; ^7^Department of Translational Proteomics, Institut de Recherches Cliniques de Montréal, Montreal, QC, Canada; ^8^Department of Medicine, Université de Montréal, Montreal, QC, Canada; ^9^Department of Biochemistry and Molecular Medicine, Université de Montréal, Montreal, QC, Canada; ^10^School of Biomedical Sciences, University of Leeds, Leeds, United Kingdom; ^11^The Children’s Hospital Research Institute of Manitoba, Winnipeg, MB, Canada; ^12^Program in Biomedical Engineering, University of Manitoba, Winnipeg, MB, Canada; ^13^Division of Experimental Medicine, McGill University, Montreal, QC, Canada

**Keywords:** GABAergic synapse, IgSF21, neurexin2α, neuroligin2, signal transduction, c-jun N-terminal kinase, CaMKII kinase, Src kinase

## Abstract

The prevailing model behind synapse development and specificity is that a multitude of adhesion molecules engage in transsynaptic interactions to induce pre- and postsynaptic assembly. How these extracellular interactions translate into intracellular signal transduction for synaptic assembly remains unclear. Here, we focus on a synapse organizing complex formed by immunoglobulin superfamily member 21 (IgSF21) and neurexin2α (Nrxn2α) that regulates GABAergic synapse development in the mouse brain. We reveal that the interaction between presynaptic Nrxn2α and postsynaptic IgSF21 is a high-affinity receptor-ligand interaction and identify a binding interface in the IgSF21-Nrxn2α complex. Despite being expressed in both dendritic and somatic regions, IgSF21 preferentially regulates dendritic GABAergic presynaptic differentiation whereas another canonical Nrxn ligand, neuroligin2 (Nlgn2), primarily regulates perisomatic presynaptic differentiation. To explore mechanisms that could underlie this compartment specificity, we targeted multiple signaling pathways pharmacologically while monitoring the synaptogenic activity of IgSF21 and Nlgn2. Interestingly, both IgSF21 and Nlgn2 require c-jun N-terminal kinase (JNK)-mediated signaling, whereas Nlgn2, but not IgSF21, additionally requires CaMKII and Src kinase activity. JNK inhibition diminished *de novo* presynaptic differentiation without affecting the maintenance of formed synapses. We further found that Nrxn2α knockout brains exhibit altered synaptic JNK activity in a sex-specific fashion, suggesting functional linkage between Nrxns and JNK. Thus, our study elucidates the structural and functional relationship of IgSF21 with Nrxn2α and distinct signaling pathways for IgSF21-Nrxn2α and Nlgn2-Nrxn synaptic organizing complexes *in vitro*. We therefore propose a revised hypothesis that Nrxns act as molecular hubs to specify synaptic properties not only through their multiple extracellular ligands but also through distinct intracellular signaling pathways of these ligands.

## Introduction

GABA-mediated inhibitory synaptic connections are very diverse due to the presence of many different types of GABA-releasing interneurons in neuronal networks (Monyer and Markram, 2004; DeFelipe et al., 2013; Huang and Paul, 2019). Another key characteristic of inhibitory synaptic connectivity is that each distinct type of GABA-releasing interneuron exhibits a different pattern of innervation onto specific subcellular compartments of target neurons (Miles et al., 1996; Freund and Katona, 2007; Muller and Remy, 2014; Tremblay et al., 2016). For example, parvalbumin (PV)-expressing basket cells innervate perisomatic regions and proximal dendrites, while somatostatin (SST)-positive Martinotti cells innervate distal dendrite regions. These complex characteristics suggest that many different types of synaptic molecular mechanisms must exist to establish the diverse and compartment-specific organization of inhibitory synapses.

Previous studies have isolated a plethora of postsynaptic organizers, molecules that trans-synaptically promote presynaptic differentiation (this ability herein called synaptogenic activity), such as neuroligin 1-4 (Nlgn1-4) (Scheiffele et al., 2000; Sudhof, 2008), leucine-rich-repeat transmembrane neuronal protein 1-4 (LRRTM1-4) (de Wit et al., 2009; Ko et al., 2009; Linhoff et al., 2009; Siddiqui et al., 2010; Roppongi et al., 2017), neurotrophin-3 receptor TrkC (Takahashi et al., 2011; Naito et al., 2017), and Slit and Trk-like proteins 1-6 (Slitrk1-6) (Takahashi et al., 2012; Yim et al., 2013). However, to date only three postsynaptic organizers have been identified that selectively regulate the development of GABA-releasing inhibitory synapses: Nlgn2 (Poulopoulos et al., 2009), Slitrk3 (Takahashi et al., 2012), and Immunoglobulin superfamily member 21 (IgSF21) (Tanabe et al., 2017). A study analyzing Nlgn2 knockout (KO) mice revealed that Nlgn2 is preferentially involved in the organization of perisomatic, rather than dendritic, inhibitory synapses in the hippocampal CA1 region (Poulopoulos et al., 2009). In contrast, we previously showed that IgSF21 is likely to be preferentially involved in the organization of dendritic rather than perisomatic inhibitory synapses in the hippocampal CA1 region (Tanabe et al., 2017). Interestingly, Nlgn2 can bind to all isoforms of neurexins (Nrxns), whereas IgSF21 can only bind to neurexin2α (Nrxn2α) (Tanabe et al., 2017). These findings raise two intriguing questions: firstly, does Nrxn2α act as a receptor for IgSF21 to mediate IgSF21-induced inhibitory synaptogenic activity; and secondly, which mechanisms are common between Nlgn2 and IgSF21 and which are unique, explaining their partially overlapping yet also distinct roles in synapse organization.

In this study, we first demonstrate that Nrxn2α acts as a high-affinity functional receptor for IgSF21 using multiple independent biochemical and cell biological experiments. We further determine a putative binding interface for IgSF21-Nrxn2α interaction using *in silico* prediction followed by experimental validation. In pharmacological experiments, we uncover that JNK underlies a common signaling pathway for Nlgn2 and IgSF21 while CaMKII and Src kinase activity underlie unique pathways for Nlgn2. Furthermore, JNK-mediated activity generates a sex-dependent regulator mechanism for Nrxn2α. This study thus provides new mechanistic insights into how synaptic organizers such as presynaptic Nrxn and its postsynaptic interaction partners, IgSF21 and Nlgn2, regulate the diversity and compartmental specificity of inhibitory synapses.

## Materials and methods

### Plasmids

The following constructs were used for cell-based assays and have previously been described (Tanabe et al., 2017): extracellularly HA-tagged mouse IgSF21 (IgSF21-HA), mouse Nrxn2α LNS1 to LNS3 domains subcloned into pDisplay (aa 29-680; pDisplay-Nrxn2α LNS1-3), soluble Fc-tagged mouse Nrxn2α ectodomain (Nrxn2α-Fc), and extracellularly HA-tagged human CD4 (HA-CD4). Novel constructs were generated for IgSF21 and Nrxn2α mutants by site-directed mutagenesis using IgSF21-HA and pDisplay-Nrxn2α LNS1-3 as templates. The IgSF21-ires-GFP construct was made by subcloning untagged rat IgSF21 into the pCAG-IRES-GFP vector (Lee A. K. et al., 2023). pNICE-NLGN2(+A) (Addgene: Plasmid #15259) was a gift from Peter Scheiffele. All constructs were verified by DNA sequencing.

### Pharmacological treatment compounds

The following chemicals were used to treat rat hippocampal neurons at the indicated concentrations: cytochalasin D (MedChemExpress, Monmouth Junction, NJ, USA; HY-N6682; 2 μM), nocodazole (MedChemExpress, Monmouth Junction, NJ, USA; HY-13520; 3.3 μM), PKI 14-22 (MedChemExpress, Monmouth Junction, NJ, USA; HY-106377A; 0.1 μM), bisindolylmaleimide I (Sigma-Aldrich; #203290; 4 μM), KN-93 (MedChemExpress, Monmouth Junction, NJ, USA; HY-15465; 5 μM), CaMKII Inhibitor XII (Sigma-Aldrich; #208923; 10 μM), PP2 (Sigma-Aldrich; #529573; 10 μM), SP600125 (MedChemExpress, Monmouth Junction, NJ, USA; HY-12041; 25 μM), and PD98059 (Selleck; #S1177; 50 μM). All compounds were dissolved in dimethyl sulfoxide (DMSO) and added to neurons to reach the indicated concentration and a final concentration of 0.2% DMSO.

### Animal and ethics statement

All animal experiments were carried out in accordance with Canadian Council on Animal Care guidelines and approved by the Institut de Recherches Cliniques de Montréal (IRCM) Animal Care Committee, the Animal Care Committee of the University of Manitoba and the University of Leeds Animal Welfare and Ethical Review Body. The Nrxn2α knockout (KO) mouse line was made in a previous study by crossing B6;129-Nrxn3^*TM*1*Sud*^/Nrxn1^*TM*1*Sud*^/Nrxn2^*TM*1*Sud*^/J mice (JAX #006377, The Jackson Laboratory, Bar Harbor, ME, USA) with the C57BL/6NCrl strain (Charles River, Margate, UK) (Dachtler et al., 2014). Genotyping by PCR used the following primers: Nrxn2α wild-type (WT) 5′ forward primer 5′-TGAGATGGAGAGCCAGACACCG-3′, Nrxn2α WT 3′ reverse primer 5′-ACAGTGCCATGGACTCAGTAGCC-3′ and Nrxn2α KO mutant 3′ reverse primer 5′-TGCATCGCATTGTCTGAGTAGGTGTC-3′. Expected PCR product sizes are 202 bp (WT) and 315 bp (KO). Mice were group-housed (two to five per cage) and maintained on a 12-h light/dark cycle.

### AlphaFold2 modeling of the IgSF21-Nrxn2α protein complex

The amino acid sequences coding for mouse IgSF21 (a.a. 25–132; UniProt ID: Q7TNR6) and mouse Nrxn2α (a.a. 29–206; Uniprot ID: E9Q7 × 7) were submitted to the AlphaFold2 server ColabFold (Mirdita et al., 2022). The number of relaxed models was set to five and the template mode was set to pdb100. Among the resulting protein complex predictions, we selected the relaxed model with the highest local structure confidence score given as pLDDT (predicted local-distance difference test). Further structural analysis and representation were performed with Pymol version 2.5.5 (PyMOL). Electrostatic potentials were mapped with the Adaptive Poisson-Boltzmann Solver (APBS) Electrostatics Plugin from Pymol (Dolinsky et al., 2004).

### Primary rodent hippocampal neuron cultures

Cultures and transfection of rat or mouse hippocampal neurons were performed essentially as described previously (Kaech and Banker, 2006; Tanabe et al., 2017; Lee A. K. et al., 2023). Briefly, hippocampal neurons from E18 rat embryos were cultured on poly-L-lysine-coated glass coverslips in neurobasal medium (Gibco; #21103-049) supplemented with NeuroCult SM1 (StemCell; #05711) and GlutaMaX (Gibco; #35050061). For Nrxn2α KO mouse neuron cultures, hippocampi from E16 mice embryo littermates derived from the crossing of Nrxn2α heterozygous mice were extracted and stored individually in tubes containing Hibernate E (BrainBits, HE500) for around 4 h until genotyping results were obtained. Meanwhile, genotyping of the mice was carried out as described above to identify WT and Nrxn2α homozygous KO littermates. Hippocampi from littermates of the same genotype were pooled, incubated in 0.25% trypsin-EDTA and dissociated, then the cells were plated onto poly-L-lysine-coated coverslips. For mouse neuron cultures, 25 mg/ml insulin (Invitrogen; #I5506) was added to the supplemented neurobasal medium. For low density cultures, the neurons were cultured on glass coverslips inverted over a feeder layer of rat astrocytes.

### Neuronal transfection and immunocytochemistry

Neuronal transfections and immunocytochemistry were performed essentially as described previously (Tanabe et al., 2017). Neuronal transfections were performed at 8 days *in vitro* (DIV) using a ProFection Mammalian Transfection System kit (Promega; #E1200) kit according to the manufacturer’s instructions. For immunocytochemistry, neurons were fixed in prewarmed 4% (v/v) formaldehyde/4% (w/v) sucrose for 12 min, permeabilized in 0.2% (v/v) Triton X-100 in phosphate-buffered saline (PBS) and blocked with 5% (v/v) normal donkey serum (NDS) and 3% (w/v) bovine serum albumin (BSA) in PBS for 1 h at room temperature. Neurons were incubated overnight at 4°C with the appropriate primary antibodies: anti-vesicular GABA transporter (VGAT) (1:500; rabbit; Synaptic Systems, Goettingen, Germany, #131003), anti-vesicular glutamate transporter 1 (VGLUT1) (1:500; guinea pig; Synaptic Systems, Goettingen, Germany; #135 304), anti-GFP (1:2,000; chicken; Abcam, Cambridge, UK; #ab13970), anti-HA (1:1,000; rat; Roche; #ROAHAHA), anti-acetylated Tubulin (1:1,000; mouse; Sigma-Aldrich; #T6793) and anti-MAP2 (1:2,000; rabbit; Abcam, Cambridge, UK; #5392). Cells were then incubated with highly cross-adsorbed Alexa dye–conjugated secondary antibodies generated in donkey toward the appropriate species (1:500; Jackson ImmunoResearch). To assess the effect of JNK inhibition on the maintenance of native synapses, neurons were treated at 14 DIV with SP600125 or DMSO as a vehicle control for 24 h.

### Artificial synapse formation assays

Cocultures of hippocampal neurons with HEK293T cells were set up as previously described (Tanabe et al., 2017; Lee H. et al., 2023). For neuron-HEK293T coculture assays, appropriate extracellularly HA-tagged plasmids were transfected in HEK293T cells using TransIT-LT1 (Mirus Bio. LLC; catalog number: MIR2305) and cultured in DMEM containing 10% (v/v) FBS. 24 h after transfection, cells were harvested by trypsinization and seeded on the neuron cultures. After 24 h of coculture, cells were fixed in prewarmed 4% (v/v) formaldehyde and 4% (w/v) sucrose in PBS for 12 min and blocked with 5% (v/v) NDS and 3% (w/v) BSA in PBS for 1 h at room temperature. Cells were incubated with anti-HA (1:1,000; rat; Roche; #ROAHAHA) overnight at 4°C to label transfected surface-expressed HA-tagged synaptic organizers. Cells were then permeabilized in 0.2% (v/v) Triton X-100 in PBS and incubated with anti-VGAT (1:500; rabbit; Synaptic Systems, Goettingen, Germany, #131003) or anti-VGLUT1 (1:500; guinea pig; Synaptic Systems, Goettingen, Germany; #135 304) overnight at 4°C. Cells were then incubated with highly cross-adsorbed Alexa dye–conjugated secondary antibodies generated in donkey toward the appropriate species (1:500; Jackson ImmunoResearch). For the pharmacological treatments, indicated compounds were added to primary hippocampal neuron cultures (14 DIV) and then 1 h later, HEK293T cells were seeded onto the neuron cultures. To investigate the effect of JNK inhibition on the maintenance of induced synapses, the transfected HEK293T cells were first cocultured with neurons for 17 h, and then the cocultures were treated with SP600125 or DMSO for another 7 h.

### *In situ* surface binding assays using soluble proteins

To produce soluble Fc-tagged proteins, plasmids encoding mouse IgSF21-Fc, Nrxn2α-Fc or Fc alone were transfected into HEK293T cells using TransIT-PRO (Mirus Bio LLC; #MIR5740) and cultured in Dulbecco’s modified Eagle’s medium (DMEM) (Gibco; #11965118) containing 10% (v/v) fetal bovine serum (FBS) (Wisent; #080-150). 24 h after transfection, the medium was replaced with serum-free AIM V synthetic medium (Gibco; #12055083). The conditioned medium was collected after 5 days. Purified human IgSF21-His (R&D Systems; #10330-S2) was purchased from R&D Systems. Protein binding assays were performed as described previously (Tanabe et al., 2017; Lee H. et al., 2023). Briefly, to assess protein binding on the surface of COS7 cells, the cells were transfected using TransIT-LT1 (Mirus Bio. LLC; #MIR2305) with plasmids encoding the target proteins fused to an extracellular HA-tag and then cultured in DMEM containing 10% (v/v) FBS. 24 h after transfection, the transfected COS7 cells were washed with extracellular solution (ECS) containing 168 mM NaCl, 2.4 mM KCl, 20 mM HEPES, pH 7.4, 10 mM D-glucose, 2 mM CaCl_2_, 1.3 mM MgCl_2_, and 100 μg/ml bovine serum albumin (BSA; Sigma, #A9647); the cells were then incubated for 1 h at 4°C with the appropriate Fc- or His-fusion proteins. IgSF21-His proteins diluted in ECS/BSA to 70 nM or Nrxn2α-Fc containing HEK293T conditioned medium were applied to COS7 cells. Cells were washed with ECS and subsequently fixed in prewarmed 4% (v/v) formaldehyde/4% (w/v) sucrose for 12 min and blocked in 5% (v/v) NDS and 3% (w/v) BSA in PBS for 1 h at room temperature. COS7 cells were incubated with anti-HA (1:1,000; rat; Roche; #ROAHAHA) and anti-6xHis (1:1,000; rabbit; Abcam, Cambridge, UK, catalog number: ab213204) without permeabilization overnight at 4°C. Cells were then incubated with highly cross-adsorbed Alexa dye–conjugated secondary antibodies generated in donkey toward the appropriate species (1:500; Jackson ImmunoResearch).

### Fluorescence imaging

Images were acquired on a Leica DM6 fluorescence microscope with 40 × 1.25 or 60 × 1.40 numerical aperture oil objectives and a Hamamatsu C11440 ORCA-Flash 4.0 camera using LasX software (Leica). Images were acquired as 16-bit grayscale. For quantification, sets of cells were stained simultaneously and imaged with identical settings.

### Image analysis

To quantify binding levels and cell surface expression levels, we measured the average intensity of each channel within the delineated COS7 or HEK293T cell area subtracted by the average intensity of the off-cell background. For *in situ* binding assays, the average intensity of bound soluble Fc- or His-proteins was normalized using the average surface intensity of the HA-tagged protein signal. COS7 cells expressing similar levels of HA-tagged proteins were selected to quantify bound soluble Fc- or His-proteins. Analyses were performed using Volocity 6.0, Excel for Microsoft 365 (Microsoft), and Prism 9 (GraphPad Software, Inc.). For the artificial synapse formation assays, HEK293T cells displaying similar surface levels of HA-tagged proteins were imaged without considering the other fluorescence channels. To assess GABAergic or glutamatergic presynaptic differentiation, the fluorescence channel corresponding to VGAT or VGLUT1 was thresholded, and the total thresholded intensity within regions positive for surface HA was measured. Analysis was performed using Metamorph 7.8 (Molecular Devices), Excel 2003, and Prism 9. For analysis in low-density neuron experiments, neurons were chosen randomly based on healthy morphology and, for transfected neurons, expression level. Images for each synaptic marker were thresholded to extract the clusters. To quantify the number of puncta, a segment of secondary dendrite per neuron or whole soma were chosen randomly based on healthy morphology and expression level, and the number of clusters per dendrite length was measured. For immunoblot quantification, band signal intensity was measured using ImageJ software and normalized to the β-actin signal or total protein (for phosphorylated targets) intensities as indicated.

### Surface biotinylation assays

Surface biotinylation assays were performed according to the kit’s instructions (Thermo Scientific, Waltham, MA, USA; #A44390). Briefly, rat hippocampal neurons were washed once with PBS and then incubated with 0.25 mg/ml EZ-Link Sulfo-NHS-SS-Biotin in PBS at room temperature for 10 min. The remaining active biotin was quenched by washing three times with ice-cold TBS. After homogenization in lysis buffer supplemented with a cocktail of protease inhibitors (Roche; #CO-RO), the biotinylated proteins were isolated using NeutrAvidin-conjugated agarose beads, resolved by SDS-PAGE, and immunoblotted with the corresponding antibodies. For pharmacological inhibition of the JNK pathway, neurons were treated at 14 DIV with SP600125 or DMSO as vehicle and surface biotinylation assays were performed 24 h post-treatment.

### Subcellular fractionation

Synaptic fractionation was carried out essentially as previously described (Carlin et al., 1980). All steps were performed on ice or at 4°C. Briefly, forebrains from 1 to 2-month-old rats or cortices from 2-month-old Nrxn2α KO and WT littermate mice were homogenized using a Douncer in homogenization buffer [0.32 M sucrose, 4 mM HEPES pH 7.4, 1 mM MgCl_2_, supplemented with a cocktail of protease (Roche; #CO-RO) and phosphatase inhibitors (Roche; #PHOSS-RO) (homogenate fraction)]. The homogenate was centrifuged at 1,500 × *g* for 15 min to collect the supernatant (postnuclear fraction). The supernatant was further centrifuged at 18,000 × *g* for 20 min and the resulting supernatant (cytosol fraction) and pellet (crude synaptosome fraction) were collected. The pellet was resuspended in homogenization buffer and loaded on a discontinuous sucrose gradient (0.85 M/1.0 M/1.2 M) and centrifuged at 78,000 × *g* for 2 h. The material at the 1.0 M/1.2 M interface was carefully collected (synaptosome fraction). Triton X-100 was added to 0.5% (v/v) and the synaptosome samples were incubated at 4°C on a rotating platform for 20 min before being centrifuged at 32,000 × *g* for 2 h. The supernatant was collected (Triton-soluble synaptosome fraction) and the pellet was resuspended in homogenization buffer, loaded on a discontinuous sucrose gradient (1.0 M/1.5 M/2.0 M) and centrifuged at 170,000 × *g* for 2 h. The material at the 1.5 M/2.0 M interface was carefully collected. Triton X-100 was added to 0.5% (v/v) and incubated at 4°C on a rotating platform for 10 min before being centrifuged at 100,000 × *g* for 20 min. The resulting pellet was resuspended in homogenization buffer (Triton-insoluble synaptosome fraction). Proteins were resolved by SDS-PAGE and immunoblotted with appropriate antibodies.

### Surface plasma resonance

Surface plasma resonance (SPR) analyses were performed using a Biacore T200 instrument (GE Healthcare, Chicago, IL, USA). Recombinant human His-tagged IgSF21 protein (R&D Systems; #10330-S2) was immobilized on carboxymethylated dextran CM5 sensor chips (Cytiva) using an amine-coupling strategy. Briefly, the sensor chip surface was activated with a 1:1 mixture of N-hydroxysuccinimide and 3-(N,N-dimethylamino)-propyl-N-ethylcarbodiimide. IgSF21 protein solution (solubilized in acetate buffer, pH 5.5) was injected at a flow rate of 20 μl/min in PBS running buffer (PBS 1X, pH 7.4, 0.025% [v/v] Tween-20) to reach a level of immobilization of 500 relative units (RU) on the CM5 sensor chip. After the immobilization step, surfaces (protein and reference) were deactivated by the injection of an ethanolamine solution to prevent further chemical reaction. Binding kinetics of recombinant human Fc-tagged NRXN2α (analyte) over the IgSF21 (target) sensor chip was evaluated in PBS running buffer (PBS 1X, pH 7.4, 0.025% [v/v] Tween-20), using a concentration series ranging from 0 to 200 nM analyte. All tests were performed at 25°C using a flow rate of 20 μl/min with a 180 sec association step (analyte) and a 240 sec dissociation step (PBS running buffer). Sensor chip surfaces were regenerated by injecting 15 μl of a 10 mM Glycine pH 3.0 solution at a flow rate of 30 μl/min. Binding sensorgrams were obtained by subtracting the signal of the reference flow cell (no protein target coupled to the sensor) from the signal obtained from the ligand flow cell (protein target coupled to the sensor). Data analysis was performed using the BIA Evaluation Software (GE Healthcare, Chicago, IL, USA). The data were fitted to a one-site Langmuir binding model using kinetic analysis and a global or local fit.

### Co-immunoprecipitation assays

Crude synaptosomal fractions were prepared essentially as described (Siddiqui et al., 2013) with some modifications. Briefly, following anesthesia of mice using isoflurane, brains were rapidly removed, rinsed with ice-cold PBS, and homogenized in homogenization buffer (320 mM sucrose, 4 mM HEPES-NaOH, pH 7.3, and protease inhibitors) in a glass Teflon homogenizer (nine strokes, 900 rpm). The homogenate was centrifuged for 10 min at 1,000 *g* to remove nuclei. The resulting pellet P1 was discarded. The supernatant S1 was centrifuged for 15 min at 12,000 *g*. The resulting pellet was resuspended in homogenization buffer and further centrifuged for 15 min at 11,000 *g*. The crude synaptosomal fraction was resuspended in lysis buffer (Complexiolyte-47 or -48 buffer, Logopharm) containing protease inhibitors, incubated for 1 h at 4°C and centrifuged at 14,000 *g* for 20 min to separate the insoluble fraction. The solubilization efficiency of synaptosomal lysates was approximately 95%. The supernatant (1.5 mg/ml protein concentration) was incubated with anti-IgSF21 (Proteintech, Chicago, IL, USA; #21465-1-AP) antibodies that were pre-conjugated to Protein G Sepharose beads for at least 12 h at 4°C. After overnight incubation of lysates with antibody-conjugated beads at 4°C, the beads were washed 4 times with Complexiolyte-47 or -48 dilution buffer and boiled in 2X Laemmli buffer (Bio-Rad, Hercules, CA, USA). To avoid detection of multiple bands due to numerous heparan sulfate modifications of Nrxns, lysates of crude synaptosomal fractions or immunoprecipitates bound to protein-G Sepharose (GE Healthcare, Chicago, IL, USA) were further incubated with a cocktail of 1 U/ml each of Heparinases I, II, III for 2 h at 37°C prior to boiling in 2X Laemmli buffer.

### Pull-down assays

For pull-down assays with highly purified proteins, 3 μg of human Nrxn2α-Fc (R&D Systems; #6636-NX), Nrxn1β-Fc (R&D Systems; #5268-NX), or Fc protein alone were incubated with 2.5 μg human IgSF21-His (R&D Systems; #10330-S2) in 500 μl of binding buffer (10 mM HEPES pH 7.4, 150 mM NaCl, 2 mM CaCl_2_, 1 mM MgCl_2_ and 0.1% Tween-20) for 1 h at room temperature. Then, 50 μl of Protein-G magnetic bead slurry (Dynabeads; Invitrogen; #10004D) was added and the samples were incubated for 1 h at 4°C on a rotating platform. Protein complexes pulled down by the Protein-G beads were purified by magnetic separation (DynaMag-2 Magnet; Invitrogen; #12321D) by washing the Protein-G beads once with binding buffer followed by four washes with PBS. Samples containing protein complexes attached to the beads were boiled in 2x Laemmli buffer containing β-mercaptoethanol, resolved by SDS-PAGE, and immunoblotted with the corresponding antibodies.

For pull-down assays coupled to mass spectrometry analysis, crude synaptosome fractions were prepared as previously described (Condomitti et al., 2018). Briefly, brains from 1-month old mice were homogenized using a Douncer with homogenization buffer [0.32 M sucrose, 4 mM HEPES pH 7.4, 1 mM MgCl_2_, supplemented with a cocktail of protease inhibitors (Roche; #CO-RO)]. The homogenate was centrifuged at 1,500 × *g* for 15 min and the supernatant was further centrifuged at 18,000 × *g* for 20 min. The pellet (crude synaptosome fraction) was resuspended in homogenization buffer containing 1.5% CHAPS detergent and incubated at 4°C for 2 h on a rotating platform before being centrifuged at 20,000 × *g* for 30 min. Protein-G magnetic beads (Dynabeads; Invitrogen; #10004D) were coated with IgSF21-Fc or control Fc proteins. Coated beads were added to the extracted crude synaptosome fractions, and the samples were incubated at 4°C overnight on a rotating platform. Bound proteins were purified by magnetic separation (DynaMag-2 Magnet; Invitrogen; #12321D) and washed three times with homogenization buffer containing 1.5% CHAPS detergent and five times with 50 mM ammonium bicarbonate (AmBic).

### Immunoblots

Protein samples were run on 8 to 12% polyacrylamide gels that were then wet-transferred onto 0.2 μm PVDF membranes (Bio-Rad, Hercules, CA, USA; #1620177). Membranes were blocked in 5% BSA diluted in tris-buffered saline with Tween-20 (TBST) (0.1% Tween-20) or 5% skim milk in TBST (0.001% Tween-20) and incubated with one of the following primary antibodies: anti-IgSF21 (1:1,000 to 1:2,000; rabbit; Proteintech, Chicago, IL, USA; #21465-1-AP), anti-PSD-95 family (1:2,000; mouse IgG2a; clone 6G6-1C9; Thermo Scientific, Waltham, MA, USA; MA1-045), anti-synaptophysin (1:10,000; mouse IgG1; clone SY38; Millipore, Kankakee, IL, USA; MAB5258), anti-Nrxn1/2/3 (1:1,000; rabbit; Millipore, Kankakee, IL, USA; #ABN161-I), anti-Nrxn1/2/3 (1:1,000; rabbit; Synaptic Systems, Goettingen, Germany; #175 003), anti-His (1:1,000; rabbit; Abcam, Cambridge, UK; #ab9108), anti-CaMKIIα [1:2,000; rabbit; Cell Signaling Technology (CST), Danvers, MA, USA; #11945], anti-CaMKIIδ (1:1,000; rabbit; LSBio, Seattle, WA, USA; #LS-C329304), anti-Src (1:1,000; mouse; Millipore, Kankakee, IL, USA; #05-184-I), anti-Fyn (1:1,000; mouse; Santa Cruz Biotechnology, Dallas, TX, USA; sc-434); anti-JNK (1:1,000; rabbit; CST; #9252), anti-phosphorylated JNK (1:1,000; rabbit; CST; #4668) and anti-β-actin (1:2,000; rabbit; Abcam, Cambridge, UK; #ab8227). Membranes were washed with TBST and incubated with horseradish peroxidase-conjugated secondary antibodies recognizing the appropriate species (1:10,000; raised in donkey, Jackson ImmunoResearch or raised in goat, Southern Biotech) or a conformation-specific mouse anti-rabbit IgG (L27A9 mAb, CST). This conformation-specific secondary antibody that does not bind to reduced and denatured IgGs was used to prevent masking of the various Nrxn isoforms by the IgG heavy and light chains of the antibody used in the co-immunoprecipitation experiments (the antibodies used in immunoprecipitation and immunoblotting are from the same host species). Signals were developed using Clarity Western ECL Substrate (Bio-Rad, Hercules, CA, USA; #1705061) or Clarity Max Western ECL Substrate (Bio-Rad, Hercules, CA, USA; #1705060) and captured by Image Lab software (Bio-Rad, Hercules, CA, USA).

### Mass spectrometry data analysis

The on-bead pulled down proteins were first incubated in 4 M urea with 50 mM AmBic for 10 min and then diluted in 2 M Urea with 50 mM AmBic for a tryptic digestion performed overnight at 37°C with agitation using 0.3 μg of Sequencing Grade Modified Trypsin (Promega; #V5111). The samples were then reduced with 13 mM dithiothreitol at 37°C for 30 min and, after cooling for 10 min, alkylated with 23 mM chloroacetamide at room temperature for 20 min in the dark. The supernatants were then acidified with trifluoroacetic acid for desalting and removal of residual detergents using an Oasis MCX 96-well Elution Plate (Waters; #186001830BA) following the manufacturer’s instructions. After elution in 90% methanol with 10% ammonium hydroxide (v/v), samples were dried with a Speed-vac, reconstituted under agitation for 15 min in 12 μL of 2% acetonitrile and 1% formic acid and loaded onto a 75 μm i.d. × 150 mm Self-Pack C18 column installed in an Easy-nLC II system (Proxeon Biosystems, Odense, Denmark). Peptides were eluted with a two-slope gradient at a flowrate of 280 nl/min. The buffers used for chromatography were 0.2% formic acid in water (solvent A) and 0.2% formic acid in acetonitrile (solvent B). Solvent B was first increased from 2 to 37% over 90 min and then from 37 to 80% over 10 min. The HPLC system was coupled to an Orbitrap Fusion mass spectrometer (Thermo Scientific, Waltham, MA, USA) through a Nanospray Flex Ion Source. Nanospray and S-lens voltages were set to 1.3-1.7 kV and 60 V, respectively. The capillary temperature was set to 250°C. Full scan MS survey spectra (m/z 360–1,560) in profile mode were acquired in the Orbitrap with a resolution of 120,000 with a target value of 3 × 10^5^. The 25 most intense peptide ions were fragmented in an HCD collision cell and analyzed in the linear ion trap with a target value of 2 × 10^4^ and a normalized collision energy at 29 V. Target ions selected for fragmentation were dynamically excluded for 15 sec after two MS2 events.

Liquid chromatography-tandem mass spectrometry (LC-MS/MS) raw files were analyzed with the Mascot search engine using the *Mus musculus* RefSeq database (version 20151112) supplemented with “common contaminants” from the Max Planck Institute^1^, the global proteome machine (GPM)^2^ and decoy sequences. The search parameters were set with trypsin specificity (two missed cleavage sites allowed), and variable modifications involving oxidation (M), deamidation (NQ) and carbamidomethyl (C) were set as fixed modifications. The mass tolerances for precursor and fragment ions were set to 10 ppm and 0.6 Da, respectively, and peptide charges of +2,+3,+4 were considered. Search results were individually processed by PeptideProphet (Keller et al., 2002), and peptides were assembled into proteins using parsimony rules as previously described (Nesvizhskii et al., 2003) by using the trans-proteomic pipeline (TPP). TPP settings were the following: -p 0.05 -x20 -PPM –d “DECOY,” iProphet option pPRIME and PeptideProphet pP. Only proteins having at least one unique peptide and an iProphet probability ≥ 0.9 were considered.

To estimate the interaction statistics, proteins having iProphet protein probability ≥ 0.9 and unique peptides ≥ 1 were submitted to SAINTexpress (version 3.6.1) (Teo et al., 2014) without control or bait compression. Potential hit proteins identified by the pulldown using IgSF21-Fc as bait were compared against pulldowns using the Fc control antibody, and interactions displaying a Bayesian false discovery rate (BFDR) ≤ 0.03 were considered as statistically significant.

### Statistical analysis of quantitative microscopy and immunoblotting experiments

Agostino’s K-squared test was used to assess normal distributions, and Bartlett’s test was used to check whether standard deviations (SDs) were significantly different across groups. To compare two groups showing normal distribution with equal variance and unequal variance, unpaired Student’s *t*-tests and Welch’s *t*-tests were used, respectively. To compare two groups without normal distribution, Mann–Whitney tests (non-parametric *t*-tests) were used. For statistical tests comparing to a hypothetical constant value, calculation of the 95% confidence interval (CI) of the mean was used. To compare three or more groups with normal distribution, Welch’s ANOVA with Dunnett’s T3 *post hoc* analysis was used. For comparisons between three or more groups without normal distribution, Kruskal–Wallis tests with Dunn’s *post hoc* analysis were used. To assess statistical differences in cumulative distribution of two groups, Kolmogorov–Smirnov tests were used. Statistical significance was examined with appropriate tests as indicated in the figure legends. All data are reported as the mean ± SEM from at least three independent experiments, and statistical significance was defined as *p* < 0.05.

## Results

### Neurexin2α acts as a high-affinity functional receptor for IgSF21

**FIGURE 1:**
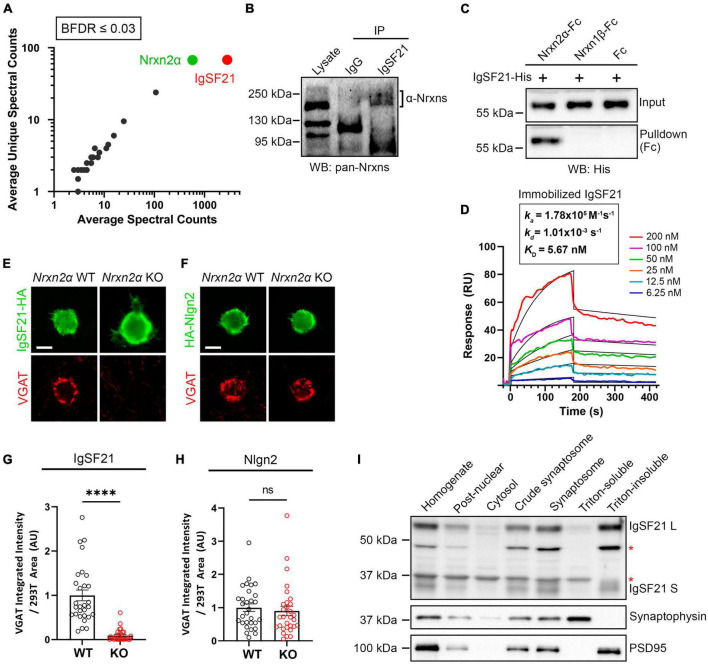
Neurexin2α acts as a high-affinity receptor for IgSF21 to mediate GABAergic presynaptic differentiation. **(A)** Identification of IgSF21-interacting proteins by unbiased pulldown assays coupled to tandem mass-spectrometry analysis. IgSF21-Fc protein coated on magnetic beads was used as bait. Following mass-spectrometry and SAINT analysis, candidates with a Bayesian false discovery rate (BFDR) of 3% or less were selected. The average of total and unique spectral counts for selected preys are shown (*n* = 2 independent experiments). **(B)** Co-immunoprecipitation assays from mouse whole brain crude synaptosomal fractions. Anti-IgSF21 co-immunoprecipitated endogenous α-Nrxns. Rabbit IgG was used as a negative control. **(C)** Pulldown assays using highly purified IgSF21-His with Nrxn2α-Fc, Nrxn1β-Fc ectodomains or Fc alone. **(D)** Surface plasmon resonance (SPR) assay using immobilized IgSF21-His and soluble Nrxn2α-Fc at a concentration range from 0 to 200 nM (colored lines). Representative sensorgrams of Nrxn2α-Fc protein fitted to a 1:1 biomolecular interaction model (black line) are shown with *K*_D_ (affinity), k_on_ (on-rate) and k_off_ (off-rate) as indicated. IgSF21 binds Nrxn2α with nanomolar affinity (*K*_D_: 5.67 nM). **(E,F)** Representative fluorescence images of VGAT accumulation induced by HEK293T cells expressing IgSF21-HA **(E)** or HA-Nlgn2 **(F)** cocultured with primary mouse hippocampal neurons derived from Nrxn2α knockout (KO) and wild-type (WT) littermates. The scale bar represents 10 μm. **(G,H)** Quantification of the total integrated intensity of VGAT accumulated on the cell surface of HEK293T cells expressing IgSF21-HA **(G)** or HA-Nlgn2 **(H)** cocultured with neurons derived from Nrxn2α WT or KO littermates, divided by the cell surface area for each HEK293T cell and normalized to the value for Nrxn2α WT. Statistical significance was examined with a Mann–Whitney test, *⁣*⁣**p < 0.0001, ns: not significant. *n* = 30 cells from three independent experiments. Data are presented as mean ± SEM. **(I)** Immunoblots of rat forebrain fractionation samples probed for IgSF21, presynaptic synaptophysin, and postsynaptic PSD95. The Triton-soluble and -insoluble fractions were enriched for synaptophysin and PSD-95, respectively. The red asterisks indicate non-specific bands.

Through *in situ* binding assays using cell lines, we have previously shown that IgSF21 binds to Nrxn2α, but not to other Nrxn isoforms, e.g., Nrxn1α, Nrxn3α, or the β-Nrxns (Tanabe et al., 2017). To confirm the binding specificity of IgSF21 to Nrxn2α in a more physiologically relevant context, we used IgSF21-Fc protein as bait in pull-down assays using mouse forebrain synaptosome samples (Figure 1A). Proteins pulled down by IgSF21-Fc were resolved by LS-MS/MS, and we unambiguously identified Nrxn2α as the top binding partner for IgSF21 (Figure 1A). These data indicate that IgSF21 has the ability to bind Nrxn2α in the brain.

Next, to address whether endogenous IgSF21 and Nrxn2α form protein complexes in the brain, we performed co-immunoprecipitation (co-IP) assays on mouse brain synaptosomes using an IgSF21 antibody for immunoprecipitation and a pan-Nrxn antibody for immunoblotting (IB) (Figure 1B). A band with an apparent molecular weight of about 200 kDa was detected in the samples immunoprecipitated by the IgSF21 antibody, but not those immunoprecipitated by rabbit IgG (Figure 1B). Together with the specificity of IgSF21 for Nrxn2α that we identified using cell-based studies (Tanabe et al., 2017), the results of the MS-coupled pull-down assay (Figure 1A) and the co-IP experiment (Figure 1B) support that native IgSF21-Nrxn2α complexes are present in mouse brain synapses.

To test whether IgSF21 and Nrxn2α engage in direct protein interaction, we performed pulldown assays using highly purified recombinant proteins. Nrxn2α-Fc, but not two negative control Fc proteins (Nrxn1β-Fc and Fc alone), pulled down IgSF21-His, revealing a direct protein interaction (Figure 1C). To gain insight into the affinity and dynamics of the IgSF21-Nrxn2α complex, we proceeded to test the binding of purified IgSF21 immobilized on a biosensor chip to soluble Nrxn2α ectodomain (0–200 nM) using surface plasma resonance (SPR). The results fit a model of bimolecular (1:1) association between an IgSF21 monomer and an Nrxn2α monomer (Figure 1D). Soluble Nrxn2α bound immobilized IgSF21 with low nanomolar concentration (*K*_D_ = 5.7 nM). Thus, Nrxn2α forms a direct, high affinity interaction with IgSF21.

IgSF21 has been shown to induce GABAergic, but not glutamatergic, presynaptic differentiation in artificial synapse formation assays. To test whether endogenous presynaptic Nrxn2α is the functional receptor for IgSF21 and is indispensable for the latter’s synaptogenic activity, we performed artificial synapse formation assays using primary hippocampal neuron cultures derived from Nrxn2α KO and WT mouse embryos (Figure 1E). IgSF21 expressed on the surface of HEK293T cells was only able to induce the accumulation of VGAT, an inhibitory presynaptic vesicle protein, on contacting axons of WT neurons and not on those of Nrxn2α KO neurons (Figure 1G and Supplementary Figure 1A). These results indicate that Nrxn2α is indispensable for the synaptogenic activity of IgSF21 and further suggest that neither other Nrxns nor any other cell surface molecules expressed on Nrxn2α KO neurons can compensate for the loss of Nrxn2α. In contrast, Nlgn2-expressing HEK293T cells induced VGAT accumulation in both WT neurons and Nrxn2α KO neurons at an equivalent level (Figures 1F, H and Supplementary Figure 1B). This is consistent with the evidence that Nrxn2α is expressed at lower abundance compared to Nrxn1α and Nrxn3α and that Nlgn2 can interact with α- and β-Nrxn1/2/3 to induce presynaptic differentiation (Treutlein et al., 2014; Schreiner et al., 2015).

In our model, IgSF21 acts as a postsynaptic organizer interacting in a *trans* manner with presynaptic Nrxn2α. To verify that IgSF21 is indeed postsynaptically expressed, we assessed its expression in subcellular fractions from rat forebrains probing the Triton-insoluble synaptosome fraction, which corresponds to the postsynaptic fraction containing PSD-95, and the Triton-soluble fraction, which contains presynaptic components including synaptophysin (Figure 1I). While there are two alternative splicing isoforms of IgSF21, a longer one (IgSF21 L) and a shorter one (IgSF21 S) (Tanabe et al., 2017), both isoforms of IgSF21 were enriched in the Triton-insoluble synaptosome fraction but absent from the Triton-soluble fraction (Figure 1I). Taken together, our results indicate that endogenous presynaptic Nrxn2α acts as a functional receptor for postsynaptic IgSF21 to mediate IgSF21-induced inhibitory presynaptic differentiation through a direct high-affinity protein interaction.

### AlphaFold2 prediction of the IgSF21-Nrxn2α complex structure

**FIGURE 2:**
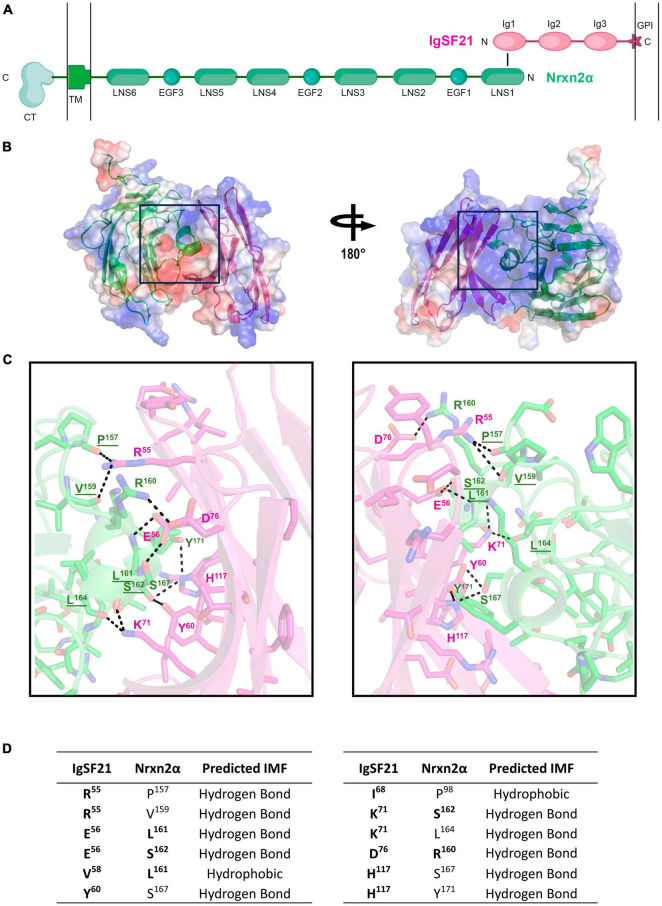
AlphaFold2 prediction of the interaction interface between the Nrxn2α LNS1 and IgSF21 Ig1 domains. **(A)** Diagram representing the domain structure of the IgSF21-Nrxn2α protein complex. IgSF21 (pink) is tethered to the cell membrane with a GPI-anchor. The LNS1 domain of Nrxn2α (green) interacts with the Ig1 domain of IgSF21. TM, transmembrane domain; CT, cytoplasmic tail. A conformation-independent representation of Nrxn2α is shown. **(B)** AlphaFold2 prediction of the LNS1 domain of Nrxn2α (green) in complex with the Ig1 domain of IgSF21 (pink). The electrostatic potential mapped onto the solvent accessible surface as calculated by PyMOL is shown together with ribbon diagrams of the proteins with positively and negatively charged regions depicted in blue and red, respectively. **(C)** Close-up view of the predicted binding interface between the Nrxn2α LNS1 and IgSF21 Ig1 domains, shown in two views rotated by ∼180°. Color code: carbon atoms in green (Nrxn2α) or pink (IgSF21), oxygen atoms in red, nitrogen atoms in blue, and sulfur atoms in yellow. Interactions discussed in the text are underlined and represent possible hydrogen bonds in the 2.7–3.8 Å range. Underlined residues are predicted to participate in hydrogen bonding by utilizing the polypeptide backbone. **(D)** Summary of residues predicted by AlphaFold2 to interact between the Nrxn2α LNS1 and IgSF21 Ig1 domains. The chemical nature of the interaction between specific residues is also indicated (falling in the range of 2.7–3.8 Å) with hydrogen bonds between both side chain and main chain atoms considered. Residues targeted for mutagenesis are indicated in bold.

So far, the tertiary structure of the IgSF21 protein or the IgSF21-Nrxn2α protein complex has not been determined. The AlphaFold Protein Structure Database (Identifier: AF-Q7TNR6-F1) and our previous study (Tanabe et al., 2017) have suggested that IgSF21 L has three immunoglobulin-like domains (Ig1-3), whereas IgSF21 S possesses only Ig1 and Ig3. According to our previous mutagenesis analysis (Tanabe et al., 2017), the IgSF21 Ig1 domain, which is common between IgSF21 L and IgSF21 S, is necessary and sufficient for Nrxn2α binding. In addition, the first laminin, neurexin and sex hormone-binding globulin-like domain (LNS1) of Nrxn2α is necessary and sufficient for IgSF21 binding. Because many Ig, Ig-like, and LNS domains exist in the Protein Databank (PDB), we chose to use AlphaFold2 (Jumper et al., 2021) to predict the tertiary structure of the IgSF21 Ig1-Nrxn2α LNS1 binding interface (Figure 2A). Five ranks were chosen for AlphaFold2 modeling of the binding interface (Ig1/LNS1) to determine the best prediction (Supplementary Figures 2A–C). Predicted local-distance difference test (pLDDT) scores were calculated for each residue of the polypeptide chains. In addition, the predicted alignment errors (PAEs) were measured between residues in the model. Rank 1 was chosen for further analysis because it contained the best chain (averaged) pLDDT and PAE scores from the 5 ranks. Our prediction modeled a binding interface in which the β-sheets (R^53^ to D^76^) of the IgSF21 Ig1 domain faced two helical protrusions (L^161^ to Y^171^) found within the loop between the 11 and 12th β-sheets (the β11-β12 loop, composed of residues from G^153^ to F^175^) of the Nrxn2α LNS1 domain (Figures 2B, C). A 4 Å cut-off value was used to assess which intermolecular forces (IMFs) could maintain the binding interface (Figures 2B, C) between IgSF21 Ig1 and Nrxn2α LNS1 in the AlphaFold2 model. A combination of many hydrogen bonds and a few hydrophobic interactions were predicted to mediate the association between IgSF21 Ig1 and Nrxn2α LNS1 (Figure 2D). For instance, IgSF21 Ig1 was predicted to utilize side chains (R^55^, E^56^, Y^60^, K^71^, D^76^, H^117^) to pack against Nrxn2α LNS1 through hydrogen bonding. On the other hand, Nrxn2α LNS1 was predicted to use side chains (R^160^, L^161^, S^162^, S^167^, Y^171^) and elements of the polypeptide backbone (P^157^, V^159^, L^161^, S^162^, L^164^) to pack against IgSF21 Ig1 via hydrogen bonding. Hydrophobic interactions between Ig1 (V^58^, I^68^) and LNS1 (P^98^, L^161^) were also possible from our predicted binding interface as listed in Figure 2D. Our binding interface model predicted more residue contacts through hydrogen bonding than hydrophobic clustering. Therefore, we concluded that perturbing the hydrogen bonding network of the interface would be most likely to destabilize the IgSF21 Ig1-Nrxn2α LNS1 complex.

**FIGURE 3:**
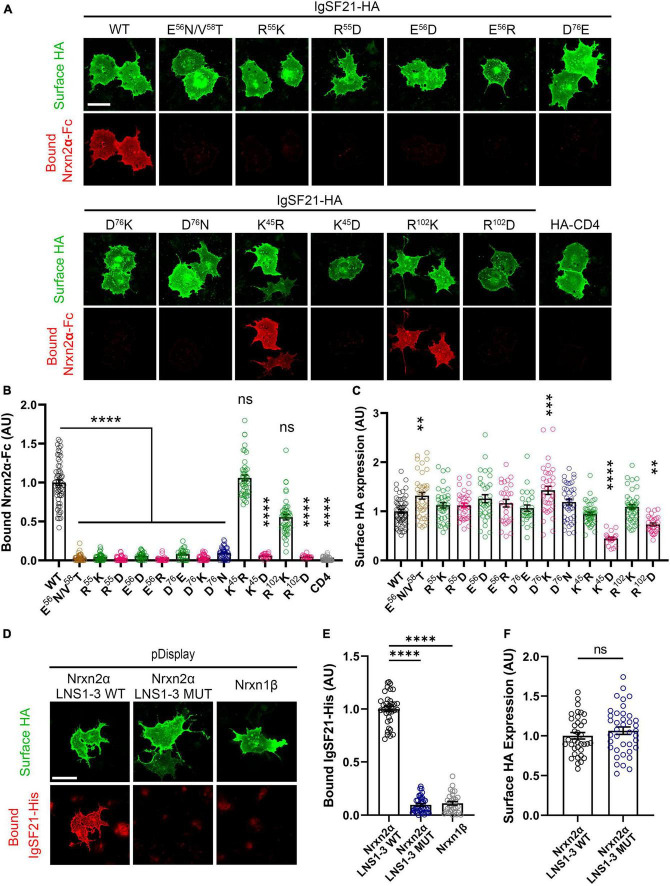
Site-directed mutagenesis at the predicted binding interface abolishes the interaction between IgSF21 and Nrxn2α. **(A)** Representative fluorescence images of soluble Nrxn2α-Fc bound to IgSF21-HA WT and mutants expressed in COS7 cells. HA-CD4 was used as a control for non-specific binding. The scale bar represents 50 μm. **(B)** Quantification of Nrxn2α-Fc bound to transfected cells described in **(A)**. The bound Nrxn2α-Fc signal was divided by the HA surface signal and normalized to the value for IgSF21 WT. The color codes in **(B)** as well as in **(C,E,F)** indicate the type of substitution: glycosylation insertion in brown, conservative replacement in green, charge reversal in magenta and charge removal in dark blue. Statistical significance was examined by a Kruskal–Wallis test with Dunn’s *post hoc* analysis for each condition compared to IgSF21 WT. ****p < 0.0001, ns: not significant. *n* ≥ 19 cells from three independent experiments. Data are presented as mean ± SEM. **(C)** Quantification of the expression of IgSF21-HA WT and mutants on the surface of COS7 cells normalized to the value for IgSF21-HA WT. Statistical significance was examined by a Kruskal–Wallis test with Dunn’s *post hoc* analysis for each condition compared to IgSF21 WT. **p < 0.01, ***p < 0.001, ****p < 0.0001, ns: not significant. *n* ≥ 19 cells from three independent experiments. Data are presented as mean ± SEM. **(D)** Representative fluorescence images of soluble IgSF21-His bound to HA-Nrxn2α LNS1-3 WT or HA-Nrxn2α LNS1-3 mutant (R^160^A/L^161^A/S^162^A) expressed in COS7 cells. Nrxn1β is used as a control for non-specific binding. The scale bar represents 50 μm. **(E)** Quantification of IgSF21-His bound to transfected cells described in **(D)**. The bound IgSF21-His signal was divided by the HA surface signal and normalized to the value for Nrxn2α LNS1-3 WT. Statistical significance was examined by Welch’s ANOVA with Dunnett’s T3 *post hoc* analysis for each condition compared to Nrxn2α LNS1-3 WT. ****p < 0.0001. *n* ≥ 30 cells from three independent experiments. Data are presented as mean ± SEM. **(F)** Quantification of the expression of Nrxn2α LNS1-3 WT and mutant on the surface of COS7 cells normalized to the value for Nrxn2α WT. Statistical significance was examined by an unpaired *t*-test. ns: not significant. *n* ≥ 30 cells from three independent experiments. Data are presented as mean ± SEM.

**FIGURE 4:**
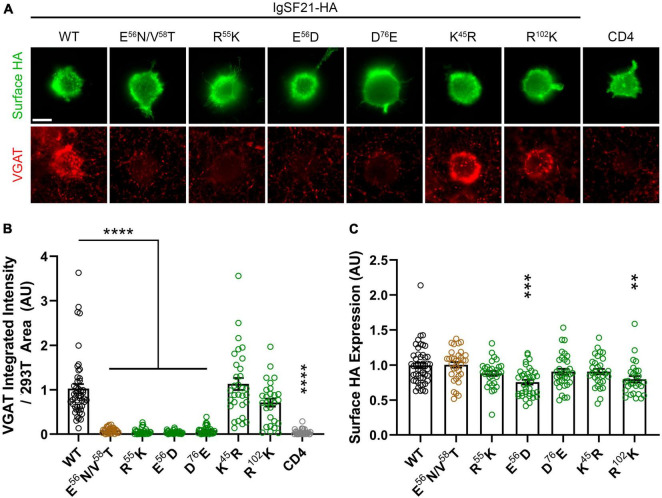
Site-directed mutagenesis at the predicted binding interface abolishes synaptogenic activity of IgSF21. **(A)** Representative fluorescence images of VGAT accumulation induced by HEK293T cells expressing IgSF21-HA WT or mutants cocultured with hippocampal neurons. HA-CD4 is used as a negative control. The scale bar represents 10 μm. **(B)** Quantification of the total integrated intensity of VGAT accumulated on the cell surface of HEK293T expressing IgSF21-HA WT and mutants or HA-CD4, divided by the cell surface area for each HEK293T cell and normalized to the value for IgSF21-HA WT. The color codes in **(B)** indicate the type of substitution: glycosylation insertion in brown and conservative replacement in green. Statistical significance was examined by a Kruskal–Wallis test with Dunn’s *post hoc* analysis for each condition compared to IgSF21 WT. ****p < 0.0001, ns: not significant. *n* ≥ 28 cells from three independent experiments. Data are presented as mean ± SEM. **(C)** Quantification of the expression of IgSF21-HA WT and mutants on the surface of HEK293T cells normalized to the value for IgSF21-HA WT. Statistical significance was examined by a Kruskal–Wallis test with Dunn’s *post hoc* analysis for each condition compared to IgSF21 WT. **p < 0.01, ***p < 0.001, ns: not significant. *n* ≥ 28 cells from three independent experiments. Data are presented as mean ± SEM.

Next, we performed cell-based validation of the IgSF21 Ig1:Nrxn2α LNS1 model predicted by AlphaFold2 (Figures 3, 4) using site-directed mutagenesis to target key residues at the interface between the two domains and disrupt the binding of the two proteins (Figure 2D and Supplementary Figure 2D). Given the exploratory nature of the IgSF21 Ig1:Nrxn2α LNS1 model, we leveraged single mutations that could have a broad impact on the interface such as charge reversal (IgSF21 Ig1 R^55^D, E^56^R, and D^76^K) or charge removal (IgSF21 Ig1 D^76^N) as well as a triple mutant (Nrxn2α LNS1 R^160^A/L^161^A/S^162^A), which would be expected to impact both electrostatic interactions and hydrogen bonds. Because charge reversal mutagenesis has the potential to disrupt overall protein structure, we also implemented conservative replacements (e.g., IgSF21 R^55^K, E^56^D, and D^76^E) which changed the shape and/or size of the side chain but maintained the charged state. To additionally engineer an impactful mutant that would be expected to be well-tolerated by the protein fold, we introduced an N-linked glycosylation site on the surface of IgSF21 Ig1 (consensus site N-X-S/T, where X is any residue except proline) in a double mutant, IgSF21 E^56^N/V^58^T. As negative controls, we selected two residues distant from the predicted binding interface (K^45^ and R^102^) for charge reversal mutagenesis (IgSF21 K^45^D and R^102^D) and conservative replacement (IgSF21 K^45^R and R^102^K).

We first tested whether and how membrane-bound HA-tagged IgSF21 WT and mutants bound soluble Nrxn2α-Fc ectodomain in *in situ* cell surface binding assays. All of the IgSF21 molecules harboring mutations at the predicted binding interface failed to interact with Nrxn2α (Figures 3A–C). Importantly, surface expression of these IgSF21 mutants was similar to that of WT, suggesting that the lack of binding of Nrxn2α on the cell surface was unlikely to be due to a lack of surface expression of the IgSF21 mutants. Intriguingly, the very conservative mutations in IgSF21 Ig1 (R^55^K, E^56^D, and D^76^E) were as effective at disrupting binding to Nrxn2α-Fc as the more drastic mutations (R^55^D, E^56^R, D^76^K, and D^76^N), indicating the very precise nature of the packing interface. In contrast, conservative replacement of the IgSF21 residues distant to the binding site (i.e., K^45^R and R^102^K) supported the predicted binding model because these replacements did not significantly affect Nrxn2α-Fc binding (Figures 3A–C). On the other hand, charge reversal substitution of these distant IgSF21 Ig1 residues (K^45^D and R^102^D) did diminish Nrxn2α-Fc binding; however, they also significantly decreased cell surface expression, suggesting that they additionally disrupted protein folding and/or protein trafficking. Our in-depth analysis of the AlphaFold2 model indicated that IgSF21 Ig1 K^45^D and R^102^D could indeed introduce unfavorable steric clashes with nearby residues (data not shown) which in turn could negatively impact their trafficking to the cell surface (Figures 3A, C). In a complementary set of binding assays, we tested whether membrane-bound mutated Nrxn2α harboring three alanine substitutions at the predicted binding interface (Nrxn2α LNS1 R^160^A/L^161^A/S^162^A) could bind to soluble IgSF21-His protein. For this test, we introduced mutations into extracellularly HA-tagged Nrxn2α encoding just the LNS1 to LNS3 domains (Nrxn2α LNS1-3) which our previous studies demonstrated are sufficient to bind to soluble IgSF21 ectodomain (Tanabe et al., 2017). Indeed, cells expressing Nrxn2α LNS1-3 WT showed significant binding of soluble IgSF21-His, while those expressing Nrxn2α LNS1-3 R^160^A/L^161^A/S^162^A construct failed to bind IgSF21-His as did Nrxn1β, whose only LNS domain is one equivalent to LNS6 and thus lacks the binding site for IgSF21-His (Figures 3D–F).

Given that IgSF21 induces presynaptic differentiation through Nrxn2α (Figures 1G, 1I), we next tested whether IgSF21 mutants that failed to bind to Nrxn2α also had impaired synaptogenic activity in artificial synapse formation assays (Figure 4). In these assays, HEK293T cells expressing either IgSF21 WT or a mutant from our panel of conservative mutants were cocultured with rat primary cultured hippocampal neurons. As expected, IgSF21 Ig1 forms introducing a glycosylation site (E^56^N/V^58^T) or carrying conservative mutations (R^55^K, E^56^D, and D^76^E) at the predicted binding interface failed to induce VGAT accumulation, while those with conservative substitutions at distant residues (K^45^R and R^102^K) induced VGAT accumulation at an equivalent level to the WT construct (Figure 4A–C). Altogether, our results show that residues predicted to form the interface between IgSF21 Ig1 and Nrxn2α LNS1 are crucial for the interaction between these two proteins and consequently for the ability of IgSF21 to induce GABAergic presynaptic differentiation.

### Exogenous IgSF21 and Nlgn2 promote GABAergic presynaptic differentiation in distinct subcellular compartments

**FIGURE 5:**
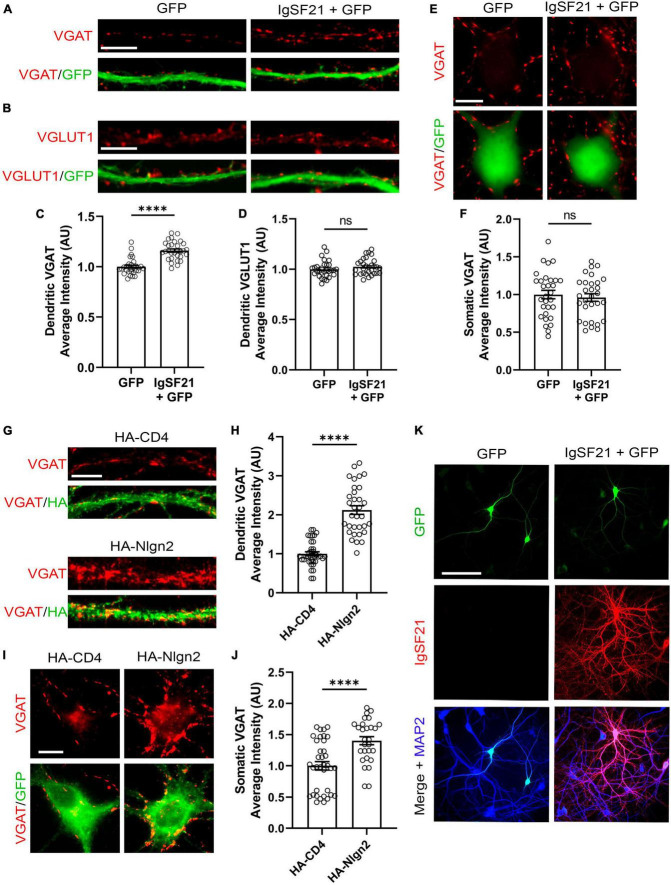
Overexpression of IgSF21 and Nlgn2 in neurons has distinct effects on somatic and dendritic GABAergic presynaptic differentiation. **(A,B)** Representative fluorescence images of VGAT **(A)** and VGLUT1 **(B)** immunoreactivity along dendrites of neurons transfected with IRES-GFP or IgSF21-IRES-GFP DNA. The scale bar represents 10 μm. **(C,D)** Quantification of the average intensity of VGAT puncta **(C)** and VGLUT1 puncta **(D)** along dendrites of IRES-GFP- or IgSF21-IRES-GFP-transfected neurons. Statistical significance was examined by a Mann–Whitney test **(C)** or an unpaired *t*-test **(D)**. ****p < 0.0001, ns: not significant. *n* = 30 cells from three independent experiments. Data are presented as mean ± SEM. **(E)** Representative fluorescence images of VGAT immunoreactivity around cell bodies of neurons transfected with IRES-GFP or IgSF21-IRES-GFP DNA constructs. The scale bar represents 10 μm. **(F)** Quantification of the average intensity of VGAT puncta around cell bodies of IRES-GFP- or IgSF21-IRES-GFP-transfected neurons. Statistical significance was examined by an unpaired *t*-test. ns: not significant. *n* = 30 cells from three independent experiments. Data are presented as mean ± SEM. **(G)** Representative fluorescence images of VGAT immunoreactivity along dendrites of neurons transfected with HA-CD4 or HA-Nlgn2. The scale bar represents 10 μm. **(H)** Quantification of the average intensity of VGAT puncta along dendrites of neurons expressing HA-CD4 or HA-Nlgn2. Statistical significance was examined by a Welch’s *t*-test. ****p < 0.0001. *n* ≥ 33 cells from three independent experiments. Data are presented as mean ± SEM. **(I)** Representative fluorescence images of VGAT immunoreactivity around cell bodies of neurons transfected with HA-CD4 or HA-Nlgn2. The scale bar represents 10 μm. **(J)** Quantification of the average intensity of VGAT puncta around cell bodies of neurons expressing HA-CD4 or HA-Nlgn2. Statistical significance was examined by a Mann–Whitney test. ****p < 0.0001. *n* ≥ 28 cells from three independent experiments. Data are presented as mean ± SEM. **(K)** Representative fluorescence images of IgSF21 and GFP immunostaining in primary rat hippocampal neurons transfected with IRES-GFP or IgSF21-IRES-GFP DNA constructs. The scale bar represents 100 μm.

Previous *in vivo* KO mouse studies have suggested that IgSF21 may preferentially regulate dendritic synaptic inhibition, whereas Nlgn2 predominantly governs somatic inhibition (Poulopoulos et al., 2009; Tanabe et al., 2017). To test whether such subcellular-compartment specificity is also maintained in an *in vitro* gain of function paradigm, we overexpressed IgSF21 and Nlgn2 in primary rat cultured hippocampal neurons and assessed pre- and postsynaptic marker immunoreactivity. IgSF21-IRES-GFP or IRES-GFP (a negative control) were transfected at 7 DIV, and immunostaining for VGAT and VGLUT1, a presynaptic marker of excitatory synapses was performed at 21 DIV. IgSF21 overexpression led to a significant increase in VGAT immunoreactivity on dendrites but not on somatic regions (Figures 5A, C, E, F). Furthermore, consistent with previous artificial synapse formation assays (Tanabe et al., 2017), IgSF21 overexpression had no effect on VGLUT1 immunoreactivity on dendrites (Figures 5B, D). In contrast, Nlgn2 overexpression led to a significant increase in VGAT immunoreactivity on both dendritic and somatic regions (Figures 5G–J). These results demonstrate that primary cultured neurons maintain molecular mechanisms that govern the subcellular-compartmental specificity of inhibitory presynaptic organization.

To test whether the specific effect of IgSF21 on dendritic, but not somatic, VGAT accumulation is due to selective dendritic expression and/or localization of IgSF21, we immunostained for IgSF21 in the transfected neurons. Interestingly, IgSF21 immunoreactivity was observed in both somatic and dendritic regions of neurons transfected with IgSF21-IRES-GFP (Figure 5K). In the neurons transfected with IRES-GFP, IgSF21 immunoreactivity was hardly detected, presumably because the IgSF21 antibody has insufficient sensitivity to detect endogenous IgSF21 (Figure 5K). These results suggest that preferential regulation of dendritic inhibition by IgSF21 is unlikely to be due to selective trafficking of IgSF21 exclusively to dendrites.

### IgSF21 and Nlgn2 synaptogenic activities rely on overlapping and distinct signaling pathways

**FIGURE 6:**
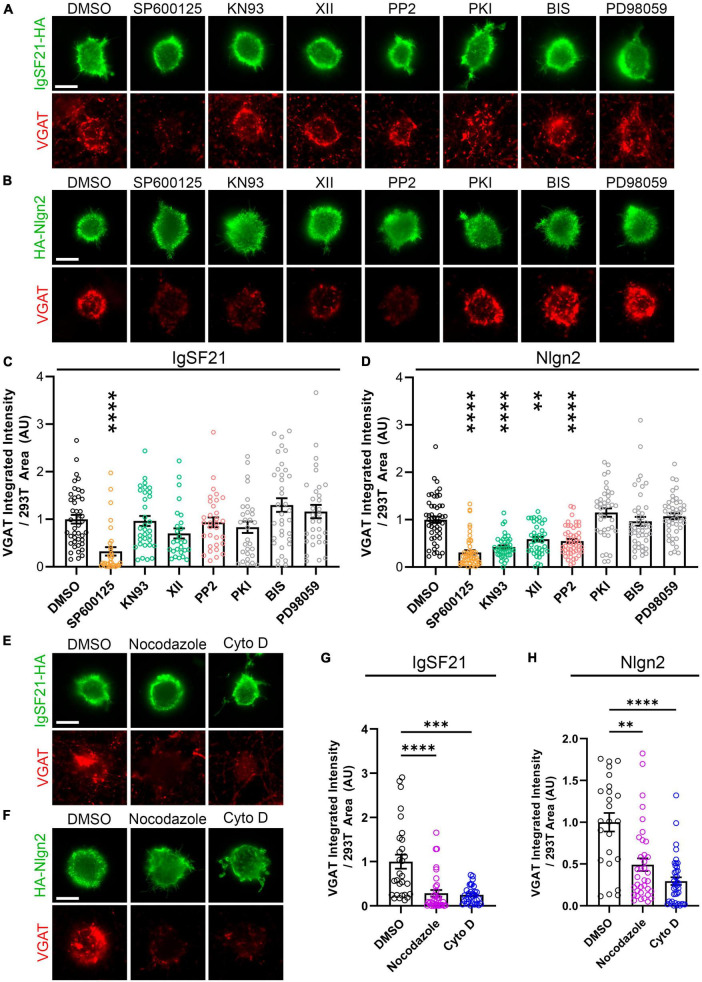
IgSF21 and Nlgn2 rely on distinct signaling pathways that partially overlap to induce GABAergic presynaptic specialization. **(A,B)** Representatives fluorescence images of VGAT accumulation induced by HEK293T cells expressing IgSF21-HA **(A)** or HA-Nlgn2 **(B)** cocultured with hippocampal neurons in the presence of pharmacological inhibitors of different signaling pathways. In all experiments, rat primary hippocampal neurons were treated at 14 days *in vitro* (DIV) with the indicated inhibitor 1 h before the seeding of transfected HEK293T cells and for another 24 h prior to immunocytochemistry. A JNK inhibitor (SP600125, 25 μM), two CaMKII inhibitors (KN93, 5 μM; XII, 10 μM), a Src inhibitor (PP2, 10 μM), a PKA inhibitor (PKI, 0.1 μM), a PKC inhibitor [Bisindolylmaleimide I (BIS), 4 μM], and a MEK1/2 inhibitor (PD98059, 50 μM) were used. DMSO is the vehicle-treated condition. The scale bar represents 10 μm. **(C,D)** Quantification of the total integrated intensity of VGAT accumulated on the cell surface of HEK293T cells expressing IgSF21-HA **(C)** or HA-Nlgn2 **(D)** and treated with the indicated inhibitor, divided by the cell surface area for each HEK293T cell and normalized to the value for the DMSO-treated control. Statistical significance was examined by a Kruskal–Wallis test with Dunn’s *post hoc* analysis for each condition compared to the DMSO-treated control. ***p < 0.001, ****p < 0.0001. *n* ≥ 27 cells from three independent experiments. Data are presented as mean ± SEM. **(E,F)** Representatives fluorescence images of VGAT accumulation induced by HEK293T cells expressing IgSF21-HA **(E)** or HA-Nlgn2 **(F)** cocultured with hippocampal neurons in presence of inhibitors of microtubule (Nocodazole, 3.3 μM) or actin filament assembly [cytochalasin D (Cyto D), 2 μM]. In all experiments, rat primary hippocampal neurons were treated at 14 DIV with the indicated compounds 1 h before the seeding of transfected HEK293T cells and for another 24 h prior to immunocytochemistry. DMSO is the vehicle-treated condition. The scale bar represents 10 μm. **(G,H)** Quantification of the total integrated intensity of VGAT accumulated on the cell surface of HEK293T cells expressing IgSF21-HA **(G)** or HA-Nlgn2 **(H)** and treated with the indicated inhibitor, divided by the cell surface area for each HEK293T cell and normalized to the value for the DMSO-treated control. Statistical significance was examined by a Kruskal–Wallis test with Dunn’s *post hoc* analysis for each condition compared to the DMSO-treated control. **p < 0.01, ***p < 0.001, ****p < 0.0001. *n* ≥ 24 cells from three independent experiments. Data are presented as mean ± SEM.

The distinct phenotypes in the above overexpression experiments between the two Nrxn ligands, IgSF21 and Nlgn2, could result from interneuron-type-dependent intracellular signaling mechanisms at GABAergic presynaptic terminals. Indeed, a previous study showed that the presynaptic phenotypes resulting from pan-Nrxn deletion were interneuron-type-specific (Chen et al., 2017). To test whether IgSF21- and Nlgn2-induced presynaptic differentiation rely on distinct intracellular signaling mechanisms, we performed artificial synapse formation assays while targeting kinase-mediated signaling pathways pharmacologically (Figure 6). Dosage of the kinase inhibitors was based on those described in Jiang et al. (2021). We found that treatment with SP600125, an inhibitor of c-jun N-terminal kinases (JNK), significantly suppressed VGAT accumulation induced by both IgSF21 and Nlgn2 (Figures 6A–D). However, treatment with KN93 or XII, two independent CaMKII inhibitors, or with PP2, an Src kinase inhibitor, did not impact the synaptogenic activity of IgSF21, while significantly blocking that of Nlgn2 (Figures 6A–D). In contrast, inhibitors of protein kinase A (PKI), protein kinase C (BIS), and MAPK/ERK kinases (PD98059) had no statistically significant effect on either IgSF21- or Nlgn2-induced presynaptic differentiation (Figures 6A–D). These results suggest that JNK signaling is widely required for inhibitory presynaptic organization, but that only a subset of synaptic organizers leverage CaMKII and Src kinases in order to regulate inhibitory presynaptic organization.

Given that JNK, CaMKII, and Src kinases are important signaling molecules involved in the regulation of the actin and microtubule cytoskeleton (Thomas et al., 1995; Shen et al., 1998; Chang et al., 2003; McVicker et al., 2015; Benoit et al., 2021), we next investigated whether the synaptogenic activity of IgSF21 and Nlgn2 was altered by the disruption of actin and microtubule cytoskeletal dynamics. We performed artificial synapse formation assays in the presence or absence of nocodazole and cytochalasin D (Cyto D) to interfere with the polymerization and stability of microtubules and actin filaments, respectively. Both compounds almost fully suppressed presynaptic differentiation induced by both IgSF21 and Nlgn2 (Figures 6E–H). Importantly, the effect of these compounds on surface expression of exogenous IgSF21 and Nlgn2 on HEK293T cells was not responsible for the loss of their synaptogenic activity (Supplementary Figures 1C, D). These results thus suggest that both synaptic organizers require intact microtubules and microfilaments to produce VGAT-positive puncta during presynaptic differentiation despite their distinct dependencies on different kinases.

**FIGURE 7:**
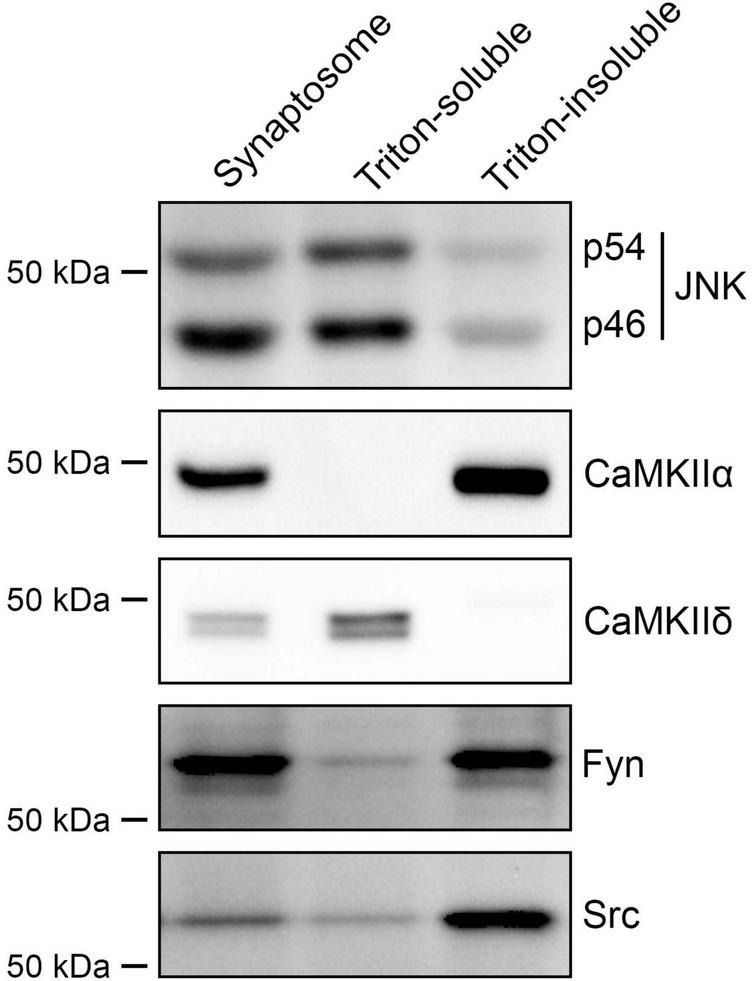
Multiple kinases underlying presynaptic specialization induced by IgSF21 and Nlgn2 display distinct subcellular localizations. Images of western blots showing rat forebrain synaptosome and the Triton-soluble and -insoluble synaptosome fractions probed for JNK1/2/3, CaMKIIα, CaMKIIδ, Fyn and Src. JNK and CaMKIIδ, but not CaMKIIα, are enriched in the Triton-soluble fraction, which represents the presynaptic side as shown in Figure 1K. Fyn and Src are slightly expressed in the Triton-soluble fraction and enriched in the Triton-insoluble fraction, which represents the postsynaptic side.

The above pharmacological results suggest that JNK, CaMKII and Src kinases act as presynaptic signaling molecules. Therefore, we next tested whether these kinases are indeed expressed at presynaptic sites (Figure 7) using Triton-soluble and -insoluble synaptosome fractions from rat forebrains, which correspond to pre- and postsynaptic fractions, respectively (Figure 1I). Both isoforms of JNK kinases, p54 and p46, were enriched in the Triton-soluble fraction, confirming the presynaptic localization of JNK kinases as previously reported (Biggi et al., 2017). Src and Fyn kinases were predominantly present in the Triton-insoluble fraction, but also in the Triton-soluble fraction, albeit with a lower enrichment. Interestingly, of the two CaMKII isoforms that we tested, CaMKIIδ was detected almost exclusively in the Triton-soluble fraction, suggesting presynaptic localization, whereas CaMKIIα was detected exclusively in the Triton-soluble fraction consistent with postsynaptic enrichment. These results indicate that presynaptic JNK regulates both IgSF21- and Nlgn2-induced inhibitory presynaptic differentiation, while presynaptic CaMKIIδ, but not postsynaptic CaMKIIα, is involved in Nlgn2-induced inhibitory presynaptic differentiation.

### JNK signaling is involved in the induction of *de novo* synapses but not the maintenance of formed synapses

**FIGURE 8:**
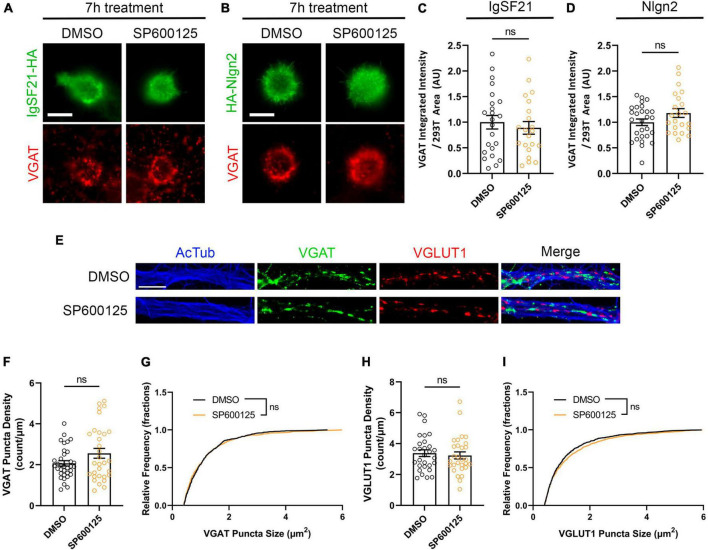
JNK signaling is not involved in the maintenance of induced or native synapses. **(A,B)** Representatives fluorescence images of VGAT accumulation induced by HEK293T cells expressing IgSF21-HA **(A)** or HA-Nlgn2 **(B)** cocultured with hippocampal neurons in the presence of a JNK inhibitor (SP600125, 25 μM). In all experiments, IgSF21-HA- or HA-Nlgn2-expressing HEK293T cells were seeded on rat primary hippocampal neurons at 14 DIV and incubated for 17 h before treatment with DMSO or SP600125 for 7 h prior to immunocytochemistry. DMSO is the vehicle-treated condition. The scale bar represents 10 μm. **(C,D)** Quantification of the total integrated intensity of VGAT accumulated on the cell surface of HEK293T cells expressing IgSF21-HA **(C)** or HA-Nlgn2 **(D)** and treated with the indicated inhibitors, divided by the cell surface area for each HEK293T cell and normalized to the value for the DMSO-treated control. Statistical significance was examined by unpaired *t*-tests. ns: not significant. *n* ≥ 21 cells from two independent experiments. Data are presented as mean ± SEM. **(E)** Representative fluorescence images of native VGAT and VGLUT1 puncta as well as acetylated α-tubulin in the presence of a JNK inhibitor (SP600125, 25 μM). In all experiments, rat primary hippocampal neurons were treated at 14 DIV with the indicated inhibitor for 24 h prior to immunocytochemistry analysis. DMSO is the vehicle-treated condition. The scale bar represents 10 μm. **(F,H)** Quantification of VGAT **(F)** or VGLUT1 **(H)** puncta density in DMSO- or SP600125-treated neurons and divided by neurite length. Statistical significance was examined by a Welch’s *t*-test **(F)** or an unpaired *t*-test **(H)**. ns: not significant. *n* ≥ 29 cells from three independent experiments. Data are presented as mean ± SEM. **(G,I)** Quantification of VGAT **(G)** or VGLUT1 **(I)** puncta size in DMSO- or SP600125-treated neurons. Statistical significance was examined by a Kolmogorov–Smirnov test. ns: not significant. *n* ≥ 29 cells from three independent experiments. Data are presented as a cumulative frequency distribution.

Having shown that JNK signaling is required for *de novo* formation of inhibitory presynaptic terminals induced by IgSF21 and Nlgn2 (Figures 6A–D), we next tested whether JNK signaling is also important for the maintenance of already formed inhibitory synapses. To do so, we first performed artificial synapse formation assays in which HEK293T cells expressing IgSF21 or Nlgn2 were cocultured with hippocampal neurons for 17 h, which is sufficient to induce presynaptic differentiation (Sudhof, 2018). Afterward, we treated the cultures with the JNK inhibitor SP600125 for another 7 h. We found that JNK inhibition had no significant effect on VGAT signals induced by either IgSF21 or Nlgn2 once these had been established (Figures 8A–D). Next, we investigated whether and how JNK signaling is involved in the maintenance of native formed synapses by treating primary rat hippocampal neurons at 14 DIV with SP600125 for 24 h. Like the results from the artificial synapse formation assays, JNK inhibition had no effect on the density and size VGAT puncta; likewise, it also had no effect on VGLUT1 puncta (Figures 8E–I). Given a previous study showing that JNK signaling is required for excitatory and inhibitory presynaptic differentiation induced by other Nrxn ligands such as Nlgn1 and LRRTM2 (Jiang et al., 2021), these results suggest that JNK signaling is involved in the differentiation, but not the maintenance, of presynaptic terminals of inhibitory synapses, and likely excitatory synapses as well.

### Nrxn2α is functionally linked with JNK signaling

**FIGURE 9:**
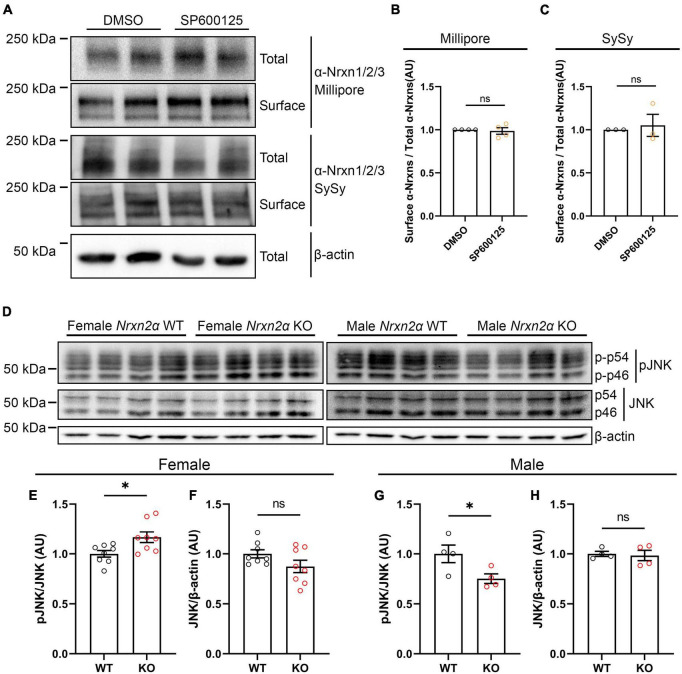
Nrxn2α KO mice display altered synaptic JNK signaling in a sex-specific manner. **(A)** Representative immunoblots of surface and total protein levels of α-Nrxns in primary rat hippocampal neurons following pharmacological inhibition of JNK signaling. Cell-surface biotinylation assays were performed at 14 DIV following 24-h treatment with 25 μM SP600125. DMSO is the vehicle-treated condition. Total and biotinylated proteins were resolved by SDS-PAGE and probed for α-Nrxn1/2/3 with two different antibodies (Millipore and Synaptic Systems (SySy)). **(B,C)** Quantification of the ratio of surface-biotinylated to total endogenous α-Nrxns probed by Millipore **(B)** or Synaptic Systems **(C)** antibodies following 24-h treatment with either DMSO or SP600125. Significance was examined by calculating the 95% confidence interval (CI) of the mean of the SP600125-treated condition. ns: not significant compared to the mean of the DMSO-treated condition. *n* ≥ 3 independent experiments. Data are presented as mean ± SEM. **(D)** Representative immunoblots of phosphorylated and total JNK protein levels in synaptosomes prepared from Nrxn2α WT and KO female (left panel) and male (right panel) mice. **(E,F,G,H)** Quantification of synaptic phosphorylated and total JNK protein levels in Nrxn2α WT and KO female (left) and male (right) mice. Phosphorylated JNK was normalized to total JNK levels, which was normalized in turn to β-actin. Significance was examined by unpaired *t*-tests. *p < 0.05, ns: not significant. Eight mice per genotype for female mice, four per genotype for male mice. Data are presented as mean ± SEM.

One explanation for the wide effects of JNK signaling on synaptogenic activity of multiple Nrxn ligands (Figures 6A–C; Jiang et al., 2021) could be a reduction of cell surface expression of endogenous Nrxns following JNK inhibition. We thus used rat hippocampal neuron cultures to perform cell surface biotinylation assays after application of SP600125 for 24 h. To detect endogenous α-Nrxns, we used two different antibodies that recognize Nrxn1/2/3 isoforms. We found that JNK inhibition did not affect total or surface expression levels of α-Nrxns (Figures 9A–C), suggesting that the inhibition of synaptogenic activity of Nrxn ligands by the JNK inhibitor is not due to a reduction in cell surface Nrxn expression but, rather, due to loss of a functional linkage between Nrxns and JNK signaling.

Next, to test whether Nrxns are functionally linked with JNK signaling, we measured the level of active phosphorylated JNK in cortical synaptosomes prepared from Nrxn2α KO mice and WT littermates. Given that previous studies showed sex-dependent phenotypes in Nrxn2α KO mice (Born et al., 2015; Dachtler et al., 2015), we prepared synaptosome samples from males and females separately. Interestingly, synaptosomes from Nrxn2α KO females displayed hyperactivation of JNK signaling (Figures 9D–F), whereas synaptosomes from Nrxn2α KO males showed hypoactivation of JNK signaling (Figures 9D, G, H). These data suggest a sex-specific functional linkage between the JNK signaling pathway and Nrxn2α. We further investigated synaptic expression level of IgSF21 in Nrxn2α KO and WT synaptosomes and found no significant difference between Nrxn2α KO and WT mice (Supplementary Figure 3). This data suggest that altered JNK activity by Nrxn2α KO may not be related to synaptic expression level of IgSF21.

## Discussion

In this study, we demonstrated that IgSF21 requires endogenous Nrxn2α to induce inhibitory presynaptic differentiation via direct high-affinity binding. Through *in silico* prediction followed by experimental validation, we further determined a possible binding model for the IgSF21-Nrxn2α complex. We also identified signaling pathways involved in the synaptogenic activity of IgSF21 and Nlgn2. Specifically, JNK is required for IgSF21 and Nlgn2 activity, while CaMKII- and Src-mediated signaling pathways are involved in Nlgn2- but not IgSF21-induced synaptogenesis. Finally, our biochemical experiments using Nrxn2α KO synaptosomes suggest the existence of a functional linkage between Nrxns and JNK signaling in synapses.

Utilizing insights into the binding between IgSF21 and Nrxn2α predicted by AlphaFold2, we successfully engineered mutations that disrupted their interaction. Notably, all of the mutations engineered at the predicted binding interface blocked the interaction between IgSF21 and Nrxn2α, a high affinity interaction (*K*_D_ = 5.7 nM). Even very conservative substitutions expected to cause only subtle changes were nevertheless still highly effective in disrupting binding between IgSF21 and Nrxn2α, suggesting that the binding interface between IgSF21 and Nrxn2α is highly optimized, yielding a high affinity interaction. Our prediction further suggests that the β11-β12 loop of the Nrxn2α LNS1 is responsible for binding with the IgSF21 Ig1 domain. Given that the β11-β12 loops of the Nrxn1α LNS2 and LNS6 domains are far from the calcium binding site of the LNS domains (Sheckler et al., 2006), the prediction of the β11-β12 loop of Nrxn2α LNS1 as a binding interface is consistent with our previous experimental evidence showing calcium-independent binding of IgSF21 with Nrxn2α (Tanabe et al., 2017). In this study, the complex was modeled only using the IgSF21 Ig1 and Nrxn2α LNS1 domains because we had previously shown that these regions are necessary and sufficient for their binding (Tanabe et al., 2017). However, other domains of IgSF21 and Nrxn2α could still be involved in IgSF21-Nrxn2α complex formation. Indeed, our previous study showed that deletion of the IgSF21 Ig3 domain significantly decreases, but does not fully disrupt, the binding of IgSF21 to Nrxn2α (Tanabe et al., 2017). Furthermore, we recently found that the three-dimensional conformation and orientation of synaptic organizers, as presented as full-length proteins within the confines of the synaptic cleft, is a critical determinant of synapse organizer complex formation (Lee H. et al., 2023). Therefore, it will be important to resolve the interaction between full length IgSF21 and Nrxn2α in future structural studies.

There are several functional differences between Nrxn2 and the other neurexins. As an extreme example, recent studies have shown a unique role of Nrxn2 in excitatory synapse function in which Nrxn2 acts as anti-synaptogenic organizer, which is at odds with the roles of Nrxn1/3 (Haile et al., 2023; Lin et al., 2023). One reason for functional differences between Nrxn2α and the other α-neurexins may be their high degree of molecular divergence. The pairwise a.a. sequence identity between Nrxn1α/2α/3α is the highest for Nrxn1α and Nrxn3α (Rowen et al., 2002; Tabuchi and Sudhof, 2002; Reissner et al., 2013) and the homology of the LNS1 domain (the region responsible for binding to IgSF21), in particular, is lower than that between the other extracellular domains of α-Nrxns. The mouse and human *NRXN2* genes are much smaller (∼110 kb) than the *NRXN1* (∼1.1 Mb) and *NRXN3* genes (∼1.6 Mb), and the intron sequences upstream or downstream of some alternatively spliced exons are highly conserved between *NRXN1* and *NRXN3* but not present in *NRXN2* (Rowen et al., 2002; Tabuchi and Sudhof, 2002; Reissner et al., 2013). The phylogenetic tree of the *NRXN* genes also suggests a possible distinct evolutionary process for the *NRXN2* gene. The molecular uniqueness of the *NRXN2* gene suggests that Nrxn2α may have acquired a distinctive interactome that governs particular types of synaptic connectivity in vertebrate brains. Indeed, IgSF21 binds to only Nrxn2α via its LNS1 domain but not to the other Nrxn isoforms, and the interaction is involved in inhibitory, but not excitatory, synapse development (Tanabe et al., 2017). In addition, both our previous *Igsf21* KO mouse study and the present *in vitro* gain-of-function study indicate that IgSF21 is involved in dendritic, rather than perisomatic, inhibitory synapse development. Thus, as a unique ligand of Nrxn2α, IgSF21 may define subcellular-compartmental specificity in diverse inhibitory synaptic connections. Further studies will be important to determine which types of GABAergic interneuron-innervated inhibitory synapses are regulated by the IgSF21-Nrxn2α complex. However, because Nrxn2α is expressed in both glutamatergic neurons and GABAergic interneurons (Ullrich et al., 1995; Uchigashima et al., 2019), its unique properties and expression patterns may not be sufficient to explain the inhibitory synapse specificity of IgSF21-Nrxn2α complex and further investigation is required to address what mechanisms underlie its inhibitory synapse specificity.

An unexpected result from our study is that the molecular mechanisms underlying inhibitory presynaptic differentiation induced by IgSF21 and Nlgn2 are different despite both being synaptic organizers that interact with Nrxn. Previous *in vivo* studies showed that Nlgn2 selectively regulates perisomatic inhibitory synapse organization (Poulopoulos et al., 2009), whereas IgSF21 seems to preferentially act in the dendritic rather than the perisomatic compartment (Tanabe et al., 2017), and we confirmed here that this is maintained in cultured neurons. Inhibitory synapses innervated by PV- and SST-positive interneurons show distinct presynaptic phenotypes upon pan-Nrxn deletion (Chen et al., 2017), and PV- and SST-positive interneurons tend to innervate perisomatic and dendritic regions, respectively (Miles et al., 1996; Freund and Katona, 2007; Muller and Remy, 2014; Tremblay et al., 2016). This raises the intriguing possibility that as of yet undefined interneuron type-specific mechanisms linked with Nrxns regulate inhibitory presynaptic selectivity. In addition to the compartmental specificity, we found that IgSF21 and Nlgn2 recruit distinct signaling pathways: while JNK regulates both IgSF21 and Nlgn2 activities, CaMKII and Src activities are involved in inhibitory presynaptic differentiation induced by Nlgn2, but not by IgSF21. Little is known about the role of presynaptic CaMKII in GABAergic synapse organization. A recent study has shown that, at Caenorhabditis elegans (*C. elegans*) neuromuscular junctions, presynaptic CaMKII (UNC-43, the *C. elegans* sole ortholog of CaMKII) trans-synaptically regulates postsynaptic GABA receptor recruitment by up-regulating presynaptic Nrxn surface delivery together with enhanced secretion of MADD-4B/Punctin (Hao et al., 2023), which makes a protein complex with Nrxn1 and Nlgn1 (Maro et al., 2015). Future studies with interneuron type-specific genetic manipulation of CaMKII and Src-mediated signaling pathways will be important to address how these pathways are linked with Nrxns in presynaptic terminals of each type of interneuron and define their GABAergic synapse specificity in developing mammalian brain circuits.

A previous study using artificial synapse formation assays in conjunction with pharmacological treatment has shown that JNK signaling pathways contribute to synaptogenic activity of Nlgn1 and LRRTM2 to promote both excitatory and inhibitory presynaptic differentiation as well as Nrxn1β-mediated glutamatergic postsynaptic assembly (Jiang et al., 2021). Our study reveals that JNK signaling also contributes to inhibitory presynaptic differentiation induced by both Nlgn2 and IgSF21. Altogether, JNK signaling may be universally involved in pre- and postsynaptic differentiation triggered by synaptic organizing complexes. Indeed, JNK signaling plays multiple roles including in regulation of the actin and microtubule cytoskeleton (Chang et al., 2003; Mengistu et al., 2011; Benoit et al., 2021), vesicle trafficking (Bharat et al., 2017; Crawley et al., 2017), SNARE complex assembly (Biggi et al., 2017), and presynaptic assembly itself (Drerup and Nechiporuk, 2013; Wu et al., 2013). Our data show that the inhibition of microtubule stabilization and actin polymerization by nocodazole and cytochalasin D, respectively, significantly impacts synaptogenic activity of IgSF21 and Nlgn2. Furthermore, we showed that JNK signaling is involved in presynaptic differentiation rather than the maintenance of formed synapses. Thus, it is possible that cytoskeletal mechanisms regulated by JNK signaling may underlie the trafficking and accumulation of synaptic vesicles induced by synaptic organizing complexes in the initial stage of presynaptic differentiation. In addition, JNK is known to interact with components of the SNARE complex including syntaxin-1/2 and SNAP25 (Biggi et al., 2017). More interestingly, inhibiting JNK activity diminishes the formation of the SNARE complex (Biggi et al., 2017). Because Nrxns indirectly interact with active zone proteins and couple calcium channels to synaptic vesicle exocytosis (Missler et al., 2003; Luo et al., 2020), JNK signaling and Nrxn-based synaptic organizing complexes may cooperatively contribute to the molecular assembly of active zone proteins including the SNARE complex and calcium channels for neurotransmitter release.

C-jun N-terminal kinases (JNKs) serve critical functions in the developing brain, influencing neural tube closure and radial migration, as well as in mature neuronal networks, regulating synaptic transmission and plasticity (Coffey, 2014). At postsynaptic sites, JNKs act downstream of ILRAPL1 to regulate PSD95 localization (Pavlowsky et al., 2010). Here, we found that loss of Nrxn2α leads to dysregulation of synaptic JNK phosphorylation without affecting synaptic expression of IgSF21. Importantly, JNK activity is regulated by neuronal activity (Nistico et al., 2015; Bharat et al., 2017), and Nrxn2α deletion impairs glutamate release and alters short-term plasticity in cortical pyramidal neurons (Born et al., 2015). It will be crucial to address synaptic mechanisms linking Nrxns to JNK signaling and their synaptic roles in both excitatory and inhibitory synapses in further future studies using Nrxn mutant mouse lines and conditional JNK KO mouse lines.

The sex-dependent dysregulation of JNK activity that we observed in Nrxn2α KO mice is intriguing. Until recently, biological sex was not considered a factor in the function of Nrxns. However, a recent meticulous study has revealed an unexpected role for Nrxn3 in the establishment of sexually dimorphic connectivity of PV-positive GABAergic terminals onto principal cells in the ventral subiculum (Boxer et al., 2021). In addition, estrogen signaling is implicated in synapse transmission and plasticity (Huang and Woolley, 2012; Tabatadze et al., 2015) and can regulate JNK activity (Srivastava et al., 1999; Adamski and Benveniste, 2005). Thus, estrogen-related signal transduction may control the functional linkage between Nrxns and JNKs and consequently lead to sex differences in modulation of neurotransmission. Interestingly, JNK1 KO mice exhibit increased explorative behaviors (Reinecke et al., 2013), reminiscent of Nrxn2 KO male but not female mice (Haile et al., 2023). On the other hand, deletion of *Nrxn2*α leads to reduced sociability in female but not male mice. Thus, JNK signaling could be a key factor that defines the sex-dependent behavioral phenotypes of Nrxn2α KO mice. In addition, given the high implication of Nrxn2 and JNKs in neurodevelopmental disorders such as autism spectrum disorders (Coffey, 2014; Khoja et al., 2023), it will be very important to gain more insights into the relationship between JNK signaling and Nrxns.

In conclusion, our study elucidates the structural and function relationship between IgSF21 and Nrxn2α and both overlapping and distinct intracellular signaling pathways of two Nrxn ligands, IgSF21 and Nlgn2, required for their roles in inhibitory presynaptic organization. Our results provide new molecular insights into the diversity and compartmental specificity of GABAergic synapse connectivity and the pathological mechanisms of neuropsychiatric disorders.

## Data availability statement

The mass spectrometry proteomics data presented in the study are deposited in the PRIDE repository, accession number PXD048927, available at http://www.ebi.ac.uk/pride/archive/projects/PXD048927.

## Ethics statement

The animal study was approved by the IRCM Animal Care Committee, the Animal Care Committee of the University of Manitoba, and the University of Leeds Animal Welfare and Ethical Review Body. The study was conducted in accordance with the local legislation and institutional requirements.

## Author contributions

NC: Conceptualization, Data curation, Formal Analysis, Investigation, Methodology, Resources, Validation, Visualization, Writing – original draft. YN: Data curation, Formal Analysis, Investigation, Validation, Visualization, Writing – review & editing. AJP: Data curation, Formal Analysis, Validation, Visualization, Writing – review & editing. NP: Data curation, Formal Analysis, Investigation, Validation, Visualization, Writing – review & editing. PN: Data curation, Formal Analysis, Investigation, Validation, Visualization, Writing – review & editing. CP: Investigation, Writing – review & editing. BF: Formal Analysis, Writing – review & editing. NY: Investigation, Writing – review & editing. JV: Formal Analysis, Investigation, Writing – review & editing. HK: Investigation, Writing – review & editing. BC: Validation, Writing – review & editing. SC: Resources, Writing – review & editing. SB: Formal Analysis, Investigation, Validation, Writing – review & editing. TS: Formal Analysis, Investigation, Validation, Visualization, Writing – review & editing. GR: Validation, Writing – review & editing. HT: Conceptualization, Data curation, Formal Analysis, Funding acquisition, Investigation, Methodology, Project administration, Supervision, Validation, Visualization, Writing – original draft.

## Footnotes

1
http://maxquant.org/downloads.htm

2
http://www.thegpm.org/crap/index.html
