# Supplementary material for: Structural and functional characterization of the IgSF21-neurexin2α complex and its related signaling pathways in the regulation of inhibitory synapse organization
Source: Front Mol Neurosci. 2024 Mar 20;17:1371145. doi: 10.3389/fnmol.2024.1371145 (PMC10989685; doi:10.3389/fnmol.2024.1371145)
Supplement: Supplementary file 1 [file Data_Sheet_1.PDF]

Supplementary Material

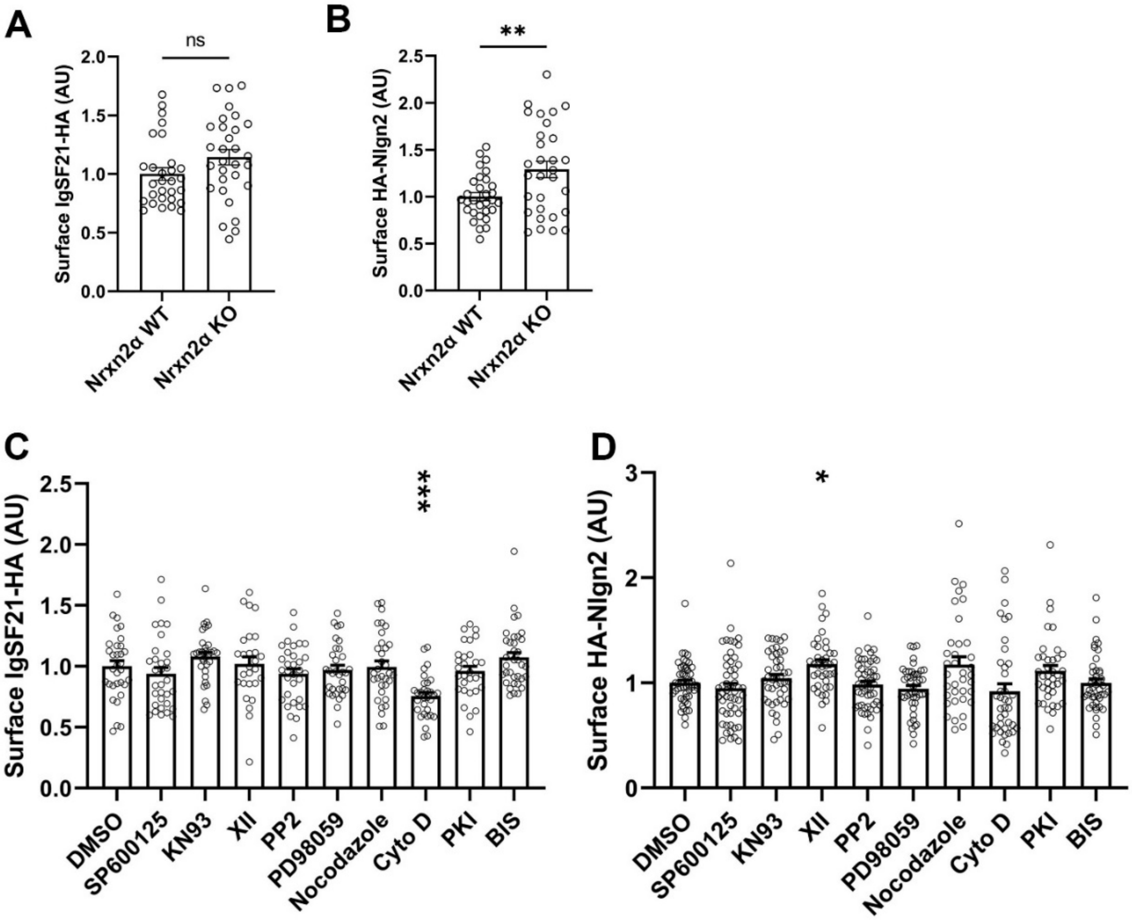

Supplementary Figure 1. Surface expression of IgSF21 and Nlgn2 in artificial synapse formation assays in Figure 1G-J and Figure 6

(A, B) Quantification of the expression of IgSF21-HA (**A**) and HA-Nlgn2 (**B**) on the surface of HEK293T cells in artificial synapse formation assays using neurons derived from Nrxn2α WT or KO pups shown in **Figure 1G-J**. The Nrxn2α WT condition was used for normalization. Statistical significance was examined by unpaired t-tests. $**p < 0.01$, ns: not significant. $n = 30$ cells from three independent experiments. Data are presented as mean ± SEM.

(C, D) Quantification of the expression of IgSF21-HA (**C**) and HA-Nlgn2 (**D**) on the surface of HEK293T cells in artificial synapse formation assays in the presence of the different signaling pathway-targeting agents shown in **Figure 6**. The DMSO-treated condition was used for normalization. Statistical significance was examined by a Kruskal Wallis test with Dunn's post hoc analysis for each condition compared to the DMSO-treated control. $*p < 0.05$, $***p < 0.001$. $n \geq 27$ cells from three independent experiments. Data are presented as mean ± SEM.

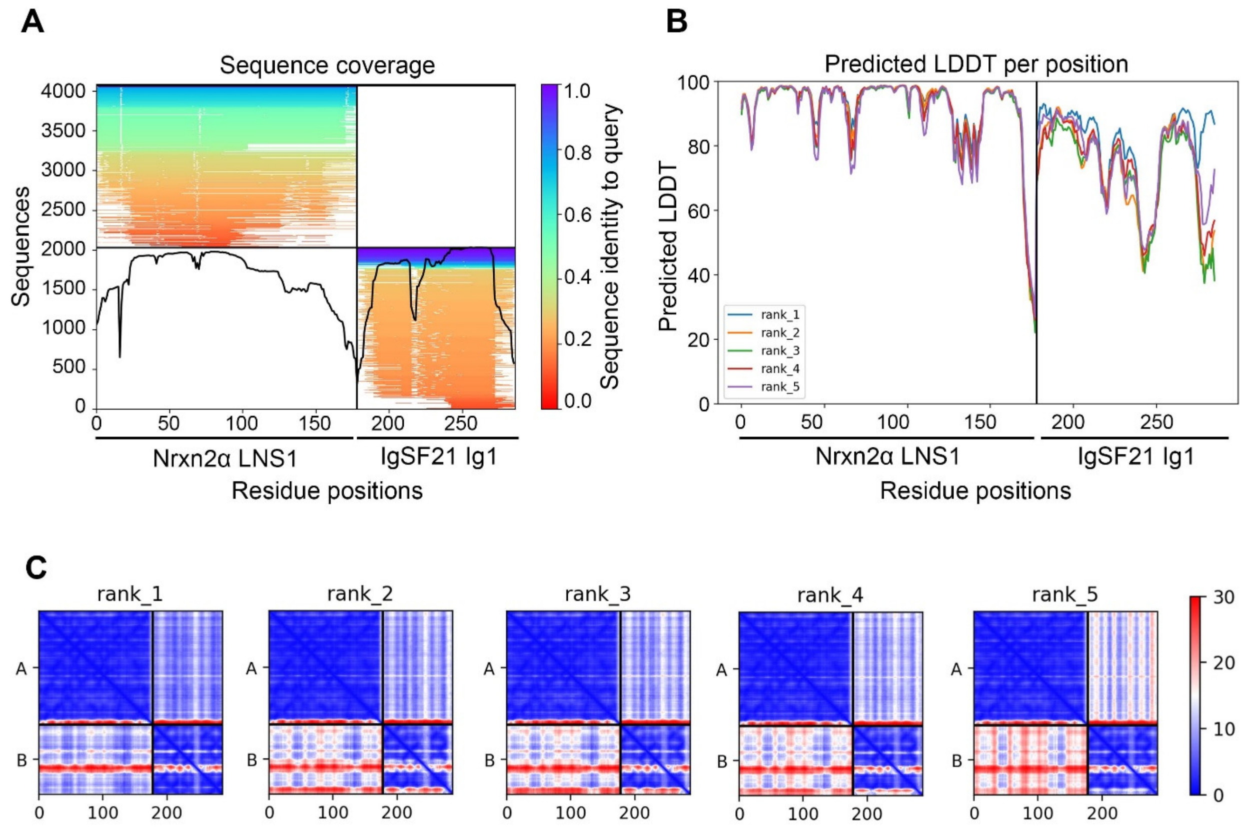

D

Mutagenesis Target	Residue	pLDDT
IgSF21	K^{45}	89.81
IgSF21	R^{55}	85.31
IgSF21	E^{56}	87.69
IgSF21	D^{76}	82.44
IgSF21	R^{102}	86.06
Nrxn2α	R^{160}	83.69
Nrxn2α	L^{161}	83.94
Nrxn2α	S^{162}	78.69

Supplementary Figure 2. AlphaFold2 prediction results for the IgSF21 Ig1 domain in complex with the Nrxn2α LNS1 domain

(A) Sequence coverage results of the multiple sequence alignment of Nrxn2α LNS1 and IgSF21 Ig1 domains based on the AlphaFold2 prediction.

(B) Predicted Local Distance Difference Test (pLDDT) results as a measure of prediction confidence of the five models of the complex between the Nrxn2α LNS1 and IgSF21 Ig1domains generated by AlphaFold2. The rank 1 model (blue) with the highest pLDDT score was selected for further analysis and is represented in Figure 2.

(C) The inter Predicted Aligned Error (PAE) results as a measure of prediction confidence of the five models of the complex between the Nrxn2α LNS1 (Chain A) and IgSF21 Ig1 (Chain B) domains generated by AlphaFold2. The PAE heatmap is shown with the indicated color scale and is represented in Å (0-30).

(D) Individual pLDDT score for each residue targeted for mutagenesis.

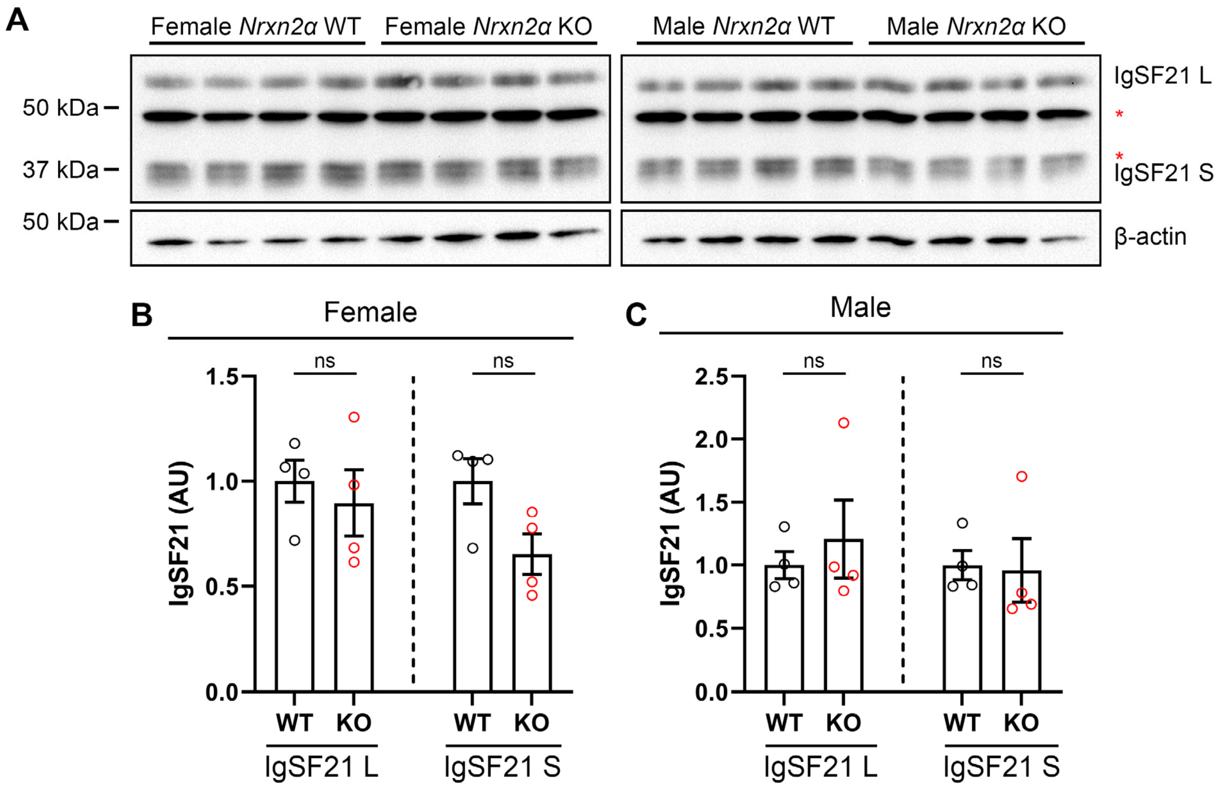

Supplementary Figure 3. Deletion of Nrxn2α does not alter the synaptic expression of IgSF21
(A) Representative immunoblots of IgSF21 protein levels in synaptosomes prepared from Nrxn2α WT and KO female (left panel) and male (right panel) mice.
(B, C) Quantification of synaptic IgSF21 protein levels in Nrxn2α WT and KO female (B) and male (C) mice. Significance was examined by unpaired t-tests. ns: not significant. Four mice per genotype for female and male mice. Data are presented as mean ± SEM.
